# Plakophilin 4 controls the spatio-temporal activity of RhoA at adherens junctions to promote cortical actin ring formation and tissue tension

**DOI:** 10.1007/s00018-024-05329-6

**Published:** 2024-07-06

**Authors:** Lisa Müller, René Keil, Markus Glaß, Mechthild Hatzfeld

**Affiliations:** Institute of Molecular Medicine, Martin Luther University Halle-Wittenberg, Charles Tanford Protein Research Center, Kurt-Mothes-Str. 3A, 06120 Halle, Germany

**Keywords:** Plakophilin 4, RhoA, Actin, Stress fibers, Tissue tension, GEFs/GAPs

## Abstract

Plakophilin 4 (PKP4) is a component of cell–cell junctions that regulates intercellular adhesion and Rho-signaling during cytokinesis with an unknown function during epidermal differentiation. Here we show that keratinocytes lacking PKP4 fail to develop a cortical actin ring, preventing adherens junction maturation and generation of tissue tension. Instead, PKP4-depleted cells display increased stress fibers. PKP4-dependent RhoA localization at AJs was required to activate a RhoA-ROCK2-MLCK-MLC2 axis and organize actin into a cortical ring. AJ-associated PKP4 provided a scaffold for the Rho activator ARHGEF2 and the RhoA effectors MLCK and MLC2, facilitating the spatio-temporal activation of RhoA signaling at cell junctions to allow cortical ring formation and actomyosin contraction. In contrast, association of PKP4 with the Rho suppressor ARHGAP23 reduced ARHGAP23 binding to RhoA which prevented RhoA activation in the cytoplasm and stress fiber formation. These data identify PKP4 as an AJ component that transduces mechanical signals into cytoskeletal organization.

**Supplementary Information:**

The online version contains supplementary material available at 10.1007/s00018-024-05329-6.

## Introduction

Epithelial integrity depends on cell–cell junctions that can resist mechanical stresses applied to tissues. Adherens junctions (AJs) bear mechanical tension and preserve tissue integrity [1, 2]. This tension arises in part from intrinsic forces, where the contractile actomyosin cytoskeleton is coupled to cadherin adhesion, and such intrinsic junctional tension occurs also in cell monolayers [3, 4]. Tension also mediates localized processes, such as cell extrusion [5, 6]. This requires that tension is not only generated and sensed at AJs but also transduced into chemical signals that alter e.g. cytoskeletal organization and junction stability [7, 8].

AJs are calcium-sensitive structures, which maintain the physical association between cells by transmembrane clusters of cadherins. The cadherin cytosolic tail domains bind to adaptor molecules including p120-catenin (p120), plakophilin 4 (PKP4, previously named p0071), β-catenin, and α-catenin, which determine cadherin stability and facilitate binding to the actin cytoskeleton. These cell–cell junction components provide not only structural integrity to the cell but also function as signaling molecules [9].

PKP4 and p120 are closely related and share roles in regulating actin-dependent cellular processes via Rho-GTPases. Generally, Rho-GTPases bind to effector molecules only in their active GTP-bound state. Their activity is controlled by guanine nucleotide exchange factors (GEFs), which activate Rho-GTPases by catalyzing GDP/GTP exchange and by GTPase-activating proteins (GAPs), which stimulate the intrinsic GTPase activity thereby inhibiting Rho-GTPases [10–12]. The spatio-temporal control of Rho-GTPase activity by GEFs and GAPs is required to maintain the organization and function of AJs. p120 inhibited RhoA and promoted Rac family small GTPase 1 (Rac1) and cell division cycle 42 (Cdc42) activation resulting in reduced cell contractility and stress fibers [13–15]. Its inhibitory effect on RhoA was mediated by an association with p190RhoGAP at the AJs [16, 17].

We have previously shown that PKP4 controls Rho-signaling during mitosis [18–20]. During cytokinesis, PKP4 stimulated RhoA activity without affecting global Rac1 or Cdc42 activities. As a result, the RhoA-dependent contractile ring formation required for abscission was disturbed in PKP4 knockdown cells resulting in failed cytokinesis and multinucleation. PKP4 associated not only with RhoA but also with the GEF epithelial cell transforming 2 (Ect2) and both, PKP4 and Ect2 were required for full activation of RhoA in vitro [20]. Moreover, PKP4 was identified as an important downstream mediator of fragile X mental retardation protein (FMRP)-dependent actin remodeling in mouse embryonic fibroblasts and in neuronal cells [21]. A role of PKP4 in regulating Rho-signaling at cell contacts has so far not been demonstrated although Ect2 was shown to localize to AJs where Ect2 activated Rho and supported junctional integrity through myosin IIA. A Centralspindlin-Ect2 complex also inhibited the junctional localization of p190B-RhoGAP (ARHGAP5), which can inactivate RhoA [22].

RhoA activates several downstream effectors, including Rho associated kinases 1 and 2 (ROCK1 and ROCK2) that phosphorylate the myosin regulatory light chains (MLC), leading to activation of myosin and generation of actomyosin contractile forces. ROCK also activates LIM-kinase (LIMK) which inhibits Cofilin’s actin depolymerization function thus stabilizing filamentous actin (F-actin). A fraction of ROCK1 was shown to co-localize with AJs and was recruited to newly forming junctions in a p120-dependent manner. ROCK1 depletion resulted in the mislocalization of the cadherin complex and of cortical actin [23]. Mechanistically, ROCK1 was necessary to stabilize GTP-RhoA and sustain junctional tension [24].

These data support the concept that junction formation and stability and the generation of intrinsic forces depend on the precise spatio-temporal regulation of RhoA activity and specific effectors. Here, we have addressed the role of PKP4 in skin keratinocytes. We show that PKP4 promotes the formation of the cortical actin ring and suppresses stress fiber formation. A general Rho activator mimicked the effect of a PKP4 knockout to a large extent. PKP4 associated with RhoA effectors as well as RhoA regulators to locally control RhoA activity and thus contractility at the cell cortex. Our data suggest that PKP4 might function as a scaffold to bring Rho-GTPases, specific regulators, and specific effectors together to control actin organization and junction stability in response to mechanical and chemical signals.

## Materials and methods

### Isolation of *Pkp4*^+*/*+^ and *Pkp4*^*−/−*^ keratinocytes

The mouse strain used for this research project, C57BL/6N-Pkp4^tm1b(EUCOMM)Wtsi^/JMmucd, RRID: MMRRC_049090-UCD, was obtained from the Mutant Mouse Resource and Research Center (MMRRC) at University of California at Davis, an NIH-funded strain repository, and was donated to the MMRRC by The KOMP Repository, University of California, Davis; Originating from Stephen Murray, The Jackson Laboratory. C57BL/6N-Pkp4^tm1b(EUCOMM)Wtsi^/JMmucd was generated by Cre-mediated excision of the parental Pkp4^tm1a(EUCOMM)Wtsi^ allele resulted in the removal of the promoter-driven neomycin selection cassette and critical exon 9, leaving behind the inserted lacZ reporter sequence. The reporter sequence was removed by breeding with B6; SJL-Tg(ACTFLPe)9205Dym/J (RRID: IMSR_JAX:003800) mice and resulting post-Cre/post-FLPe mice were backcrossed onto the C57BL/6 J (RRID: IMSR_JAX:000664) background for 10 backcross generations. Thereafter Pkp4^±^ siblings were intercrossed to generate Pkp4^+/+^ and Pkp4^−/−^ animals.

Primary keratinocytes of Pkp4^+/+^ and Pkp4^−/−^ mice were isolated from newborn pups, essentially as described in Rietscher et al. [25]. Briefly, the dissected skin was incubated with 5 mg/ml Dispase (Roche diagnostics, Mannheim, Germany) in phosphate buffered saline (PBS) overnight at 4 °C to separate the epidermis from the dermis. Subsequently, the epidermal sheet was incubated in trypsin solution (0.025% trypsin in PBS/ 0.02% EDTA) for 10 min at 37 °C to liberate the keratinocytes [hereafter PKP4-wildtype (WT) and PKP4-knockout (PKP4-KO)].

### Cell lines, cell culture, and treatments

HEK293T cells were grown in Dulbecco´s modified Eagle´s medium [DMEM, 4.5 g/l high glucose, 1 mM sodium pyruvate, 1 mM glutamate, 10% (v/v) fetal calf serum (FCS)] at 37 °C in 5% CO_2_ and 90% humidity. Mouse keratinocytes were grown on 15 µg/ml collagen I (Corning, Glendale, Arizona, USA) in low calcium medium [LCM; DMEM/Ham’s F12 medium containing 50 μM CaCl_2_, 10% (v/v) Ca^2+^-free FCS, 1 mM sodium pyruvate, 1 mM glutamate, 0.18 mM adenine, 0.5 μg/ml hydrocortisone, 5 μg/ml insulin, 10 ng/ml EGF, 100 pM cholera toxin, 1 mg/ml D-( +)-glucose] at 32 °C in 5% CO_2_ and 90% humidity.

To generate PKP4-KO keratinocytes expressing EGFP-PKP4-WT (PKP4-KO + PKP4 rescue cells) and WT cells expressing EGFP (GFP cells), HEK293T cells were co-transfected by CaPO_4_ precipitation with the packaging plasmids pMD2.G (Addgene plasmid #12259; gift from Didier Trono), psPAX2 (Addgene plasmid #12260; gift from Didier Trono), and lentiviral expression vector pLVX-IRES-puro encoding either EGFP or human PKP4 N-terminally taged with EGFP. Lentiviral particles were purified 48 h after transfection using Lenti-X concentrator (Takara Bio Inc., Kusatsu, Shiga, Japan) according to the manufacturer´s protocol. Keratinocytes were incubated with the lentiviral particles for 24 h and subsequently selected using puromycin (1 µg/ml) to obtain stable cell lines. The resulting cell lines were analyzed by fluorescence microscopy and western blotting to monitor expression of transgenes.

To induce the differentiation of keratinocytes, LCM was supplemented with 1.2 mM CaCl_2_ (high calcium medium; HCM).

For activation of Rho- or Rac/Cdc42-GTPases, keratinocytes were treated with 5 µg/ml Rho activator II (Cytoskeleton) or 5 unit/ml Rac/Cdc42 activator II (Cytoskeleton) for 2 h, respectively. PBS was used as a control treatment.

To analyze the stability of intercellular junctions, keratinocytes were treated with 3.3 mM ethylene glycol-bis-(2-aminoethylether)-N,N,N′,N′-tetraacetic acid (EGTA) for 3 h.

### Transfections

Transfection of plasmid DNA was performed using Xfect™ (Takara Bio Inc.) according to the manufacturer’s instructions. Keratinocytes were grown in LCM for 24 h, incubated with the plasmid-Xfect mixture for 4 h, and maintained in HCM for an additional 24 h.

The siRNA pools (defined pools of 30 selected siRNAs, Table S1) were obtained from siTools Biotech GmbH (Martinsried, Germany) and transfected using Lipofectamine® RNAiMax (Thermo Fisher Scientific, Waltham, MA, USA) according to the manufacturer’s instructions. Keratinocytes transfected in suspension with 2 pmol of the respective siRNA pools were switched to HCM at 48 h after transfection and kept in HCM for another 24 h.

### Plasmids and cloning

Human cDNAs of mCitrine-YFP-ARHGAP23 and mCitrine-YFP-ARHGEF2 were a kind gift from Oliver Rocks [12]. RhoA-CA(Q63L) and RhoA-CN(T19N) were a gift from Gary Bokoch [26] (Addgene plasmids #12968, #12967). EGFP-RhoA Biosensor was a gift from Michael Glotzer [27] (Addgene plasmid #68026). Human cDNA of PKP4-WT was subcloned into pLVX-IRES-puro (Takara Bio Inc.) containing an EGFP ORF. Vectors for the production of lentiviral particles pMD2.G and psPAX2 were a gift from Didier Trono (Addgene plasmids #12259, #12260). All constructs were validated by sequencing.

### Protein extraction

For protein expression analysis, keratinocytes were lysed in sodium dodecyl sulfate (SDS) buffer [2.5% (v/v) SDS pH 7.5, 1 mM EDTA, 100 mM HEPES; supplemented with 1 × Halt™ protease and phosphatase inhibitor cocktail (Thermo Fisher Scientific)] and centrifuged for 15 min at 13,000 g. The protein concentration was determined using the Pierce™ BCA Protein Assay Kit (Thermo Fisher Scientific) according to the manufacturer`s protocol. Benzonase (Santa Cruz Biotechnology, Dallas, Texas, USA) and SDS-polyacrylamide gel electrophoresis (SDS-PAGE) loading buffer [250 mM Tris/HCl (pH 6.8), 30% (v/v) glycerol, 0.25% (w/v) bromophenol blue, 10% (v/v) β-mercaptoethanol, 8% (v/v) SDS] were added. Samples were heated to 95 °C for 5 min, separated by SDS-PAGE, and analyzed by western blotting.

### Immunoprecipitation

All steps of immunoprecipitation (IP) reactions were performed on ice. Cells were lysed in IP-buffer [20 mM Tris–HCl pH 7.5, 137 mM NaCl, 2 mM EDTA, 10% (v/v) glycerol, 1% (v/v) NP-40; supplemented with 1 × Halt™ protease and phosphatase inhibitor cocktail]. Lysates were cleared by centrifugation for 15 min at 4 °C and 13,000 g. One-sixth of the lysate was mixed with SDS-PAGE loading buffer and stored at −20 °C as an input control. The residual lysate was incubated with anti-ARHGAP23 (Affinity Biosciences), anti-ARHGEF2 (Cell Signaling Technology), or anti-PKP4 (Peptide Specialty Laboratories) antibody overnight at 4 °C on an overhead rotator. Normal rabbit IgG (Santa Cruz) was used as an isotype control. Protein A agarose beads (Thermo Fisher Scientific) were washed in IP buffer and added to the lysate for 1 h at 4 °C on an overhead rotator. Lysates were centrifuged for 3 min at 4 °C and 4,000 g, washed three times in IP buffer, and bound proteins eluted in SDS-PAGE loading buffer.

For GFP-Trap experiments, cells expressing a GFP-tagged protein were treated as described above. The obtained cell lysates were incubated with GFP-Trap Agarose (ChromoTek, Planegg, Germany) for 1 h at 4 °C on an overhead rotator, washed as described above, and eluted in SDS-PAGE loading buffer. In either case, input and eluate samples were separated by SDS-PAGE and analyzed by western blotting.

### SDS-PAGE and western blotting

Equal amounts of protein were separated by SDS-PAGE. Proteins were transferred to Amersham™ Protan™ nitrocellulose blotting membranes (pore size 0.2 µm, Th. Geyer, Renningen, Germany) using Mini Trans-Blot cells (Bio-Rad Laboratories, Hercules, California, USA). After transfer, membranes were stained in Ponceau S solution [0.2% (w/v) Ponceau S, 3% (w/v) TCA, 3% (w/v) sulfosalicylic acid] for 5 min, washed in distilled water, and documented using a scanner. Membranes were cut, destained in Tris-buffered saline with Tween20 (TBST), blocked with 3% (w/v) skimmed milk/TBST or 3% (w/v) bovine serum albumin (BSA)/TBST, and subsequently probed overnight with the appropriate primary antibodies as listed in Table S2. Membranes were washed three times with TBST and incubated for 1 h with the appropriate horseradish peroxidase-conjugated secondary antibodies (Dianova, Hamburg, Germany, see Table S3). Membranes were treated with ECL Western Blotting Substrate [equal parts chemiluminescence solution 1 (100 mM Tris/HCl [pH 8.5], 25 mM luminol, 0.4 mM coumaric acid) and 2 (100 mM Tris/HCl [pH 8.5], 0.02% [v/v] H_2_O_2_)] or Western Blot Ultra-Sensitive HRP Substrate (Takara) and chemiluminescence was detected using a Fusion-SL 3500WL imaging system (Peqlab, Erlangen, Germany). If staining with additional antibodies was required, the membranes were washed three times in TBST for 10 min each, incubated in stripping buffer [0.2 M glycine, 0.05% tween-20 (pH 2.5)] for 1 h, washed again in TBST (3 × 10 min), blocked, and treated with antibodies as described above. Glyceraldehyde-3-phosphate dehydrogenase (GAPDH) or Ponceau S staining was used as the loading control.

### Immunofluorescence analysis and image processing

Cells were grown on 12-mm collagen I-coated glass coverslips and fixed for 10 min in methanol at −20 °C or for 20 min in 3.7% (w/v) formaldehyde in PBS on ice or room temperature, permeabilized in detergent buffer [100 mM PIPES (pH 6.9), 4 M glycerol, 2 mM EDTA, 1 mM EGTA, 0.5% (v/v) Triton X-100] for 15 min at room temperature, and blocked in 1% (w/v) BSA/PBS for 30 min at room temperature. Primary antibodies were diluted in blocking solution and incubated overnight at 4 °C in a humid chamber. The next day, coverslips were washed in PBS, briefly blocked, and incubated for 1 h at room temperature with the fluorophore-conjugated secondary antibody. DNA was stained with Hoechst 33,342 (Thermo Fisher Scientific). Antibodies used for immunofluorescence are listed in Table S2 and Table S3. Coverslips were mounted in Mowiol [5% (w/v) Mowiol, 30% (v/v) glycerol, 0.25% (w/v) 1,4-diazabicyclo[2.2.2]octane (DABCO)]. Images were taken using a Nikon Eclipse E600 microscope, a CCD camera, and a Plan APO 60xA/1.40 oil objective controlled by the NIS-Elements AR software (version 4.12.00). For comparisons between WT, PKP4-KO, and Rescue cells, samples were treated in parallel and images captured at the same exposure times. Fiji software was used for image processing [28].

### Epithelial sheet assay (dispase assay)

For analysis of intercellular adhesion, WT and PKP4-KO keratinocytes were kept in HCM for 24 h or 72 h before incubation with 2.4 U/ml dispase II (Roche Diagnostics, Indianapolis, IN, USA) in DMEM/Ham’s F12 medium supplemented with 1.2 mM Ca^2+^ and 25 mM HEPES for 30 min at 37 °C. After detachment, monolayers were kept for additional 30 min in DMEM/Ham’s F12 medium containing 1.2 mM Ca^2+^, 25 mM HEPES and 3.3 mM EGTA before submitting to mechanical stress on an orbital shaker at 750 rpm. For analysis of intercellular cohesion in combination with knockdown, WT and PKP4-KO keratinocytes transfected with non-targeting (Ctrl), ARHGEF2-, or ARHGAP23-directed siRNA-pools, were switched to HCM at 48 h after transfection and kept in HCM for another 24 h before incubation with 2.4 U/ml dispase II. Floating monolayers were treated as above. Images were taken using a Sony DSC-H300 camera. For image processing, counting of fragments, and measuring monolayer size, the ImageJ tools “Cell counter” and “Polygon selection” were used.

### G-actin/F-actin ratio assay

The G-Actin and F-Actin ratio was implemented through the G-Actin/F-Actin in vivo assay kit (Cat. #BK037, Cytoskeleton Inc.). Briefly, the keratinocytes were washed two times in PBS before lysis with warm LAS2 buffer (1 ml of lysis and F-actin stabilization buffer, 10 µL of ATP stock solution (100 mM), 10 µL of 100 × protease cocktail inhibitor) and homogenized using a 25G syringe. The lysates were kept at 37 °C for 10 min and then centrifuged at 350 × g in a tabletop microfuge at room temperature for 5 min to pellet unbroken cells. The obtained supernatants were transferred into ultracentrifuge tubes and centrifuged at 100,000 × g, 37 °C for 1 h to pellet F-actin and leave G-actin in the supernatant. The obtained supernatants were transferred to fresh tubes designated as G-actin samples. F-actin depolymerization buffer (supplied in the kit) was added to the remaining pellets and incubated on ice for 1 h to allow for actin depolymerization. For SDS-PAGE, pellet (F-actin) and supernatant (G-actin) samples were mixed with SDS-PAGE loading buffer and processed for western blotting.

### RhoA activation assay

7.5 × 10^6^ WT, PKP4-KO, or Rescue keratinocytes were seeded onto collagen I-coated 15 cm dishes in LCM. After 24 h, the medium was changed to HCM for another 24 h. Subsequently, keratinocytes were lysed using 750 µl Mg^2+^ lysis/wash buffer (MLB; 25 mM HEPES, pH 7.5, 150 mM NaCl, 1% Igepal CA-630, 10 mM MgCl_2_, 1 mM EDTA and 2% glycerol) and the Rho Activation Assay was performed using Rhotekin Rho-binding domain agarose (Merck, Darmstadt, Germany) according to the manufacturer´s protocol. Amounts of Rhotekin-agarose bound RhoA were analyzed by western blotting using a RhoA Rabbit mAb (Cell Signaling Technology).

### RNA expression

For validation of knockdown efficiencies, keratinocytes transfected in suspension with 2 pmol of the respective siRNA pools were switched to HCM at 48 h after transfection and kept in HCM for another 24 h. Cells were homogenized in Trizol and RNA isolated by phenol/chloroform extraction and isopropanol precipitation. For cDNA synthesis, 1 µg of total RNA served as the template using SuperScript® II Reverse Transcriptase (Thermo Fisher Scientific) and random hexamer primers following the manufacturer’s protocol. Real-time PCR was performed with primaQUANT 2 × qPCR CYBR Green MasterMix w/o ROX (Steinbrenner-Laborsysteme GmbH, Wiesenbach, Germany) using a LightCycler 480 II Real Time PCR system (Roche, Basel, Switzerland) with the following PCR conditions: 95 °C for 15 min followed by 40 cycles of 95 °C for 15 s, 62 °C for 15 s, and 72 °C for 20 s. Primer pairs were selected using Primer Blast (https://www.ncbi.nlm.nih.gov/tools/primer-blast/). The primer sequences are listed in Table S4.

### RNA sequencing

Keratinocytes were grown for 24 h or 72 h in LCM or HCM. Cells were homogenized in Trizol and RNA isolated by phenol/chloroform extraction and isopropanol precipitation. Library preparation (Poly(A) tail RNA selection) and strand-specific RNA sequencing (total RNA amount ≥ 2 µg) was performed by LC Sciences (Houston, USA) on a Novaseq6000 system (Illumina, San Diego, USA), producing 149 bp long paired-end sequencing reads at an average depth of 2 × 19.7 million reads per sample.

### RNA-seq data processing

Quality of the raw fastq files was assessed using FastQC (https://www.bioinformatics.babraham.ac.uk/projects/fastqc/). Sequencing reads were aligned to the murine reference genome (UCSC mm39) concatenated with the GFP-PKP4 sequence using HiSat2 v2.1.0 [29]. Alignments in the obtained bam files were sorted, indexed and secondary alignments were filtered out using samtools v1.10 [30]. FeatureCounts v2.0.0 [31] was used for summarizing gene-mapped reads. Ensembl (GRCm39.105; [32]) was used as annotation basis. Differential gene expression was determined using the R package edgeR v3.42.4 utilizing trimmed mean of M-values normalization [33, 34]. A false discovery rate (FDR) adjusted p-value below 0.05 was considered significant for differential gene expression.

### Gene set enrichment analysis

Gene set enrichment analysis (GSEA) was performed using the R-package clusterProfiler (v 4.8.2; [35] and MSigDB mouse gene sets (v2023.1; [36]) utilizing the fgsea algorithm and setting the exponent parameter to 0 for unweighted analyses of log2 fold change sorted gene lists obtained from differential gene expression analyses.

### Western blot quantification

For quantification of western blots, the ImageJ tool “Gel Analysis” was used. All signals were normalized to the internal loading control GAPDH. If GAPDH expression varied due to experimental conditions, Ponceau S staining was used as the loading control. Details concerning normalization procedures are given in the figure legends.

### Quantification of immunofluorescence

To determine the enrichment factors in the nucleus and cytoplasm, fluorescence intensities were measured as described previously [37]. The quantification of fluorescence intensities at lateral and tricellular contacts have been described previously [38].

### Statistical analysis

Statistical analysis and plot preparation were performed using Graphpad Prism Software (version 8.3). All individual data points are shown in the plots. Boxplots display the first to third quartile; whiskers extend to the minimum and maximum. For two independent data sets, significant differences were determined by a two-tailed Student’s t-test. To compare more than two independent data sets with normal distribution, one-way analysis of variance (ANOVA) followed by a Tukey’s multiple comparison test was used.

## Results

### PKP4 regulates actin- and adhesion-associated pathway activities

The dynamic connection of AJs to the actin cytoskeleton is tightly regulated by Rho-signaling. When misregulated, abnormal junctional actin polymerization can drive pathological conditions including cancer, vascular and neurodegenerative diseases [39]. As a novel model system to study how PKP4 affects the cortical actin cytoskeleton via Rho signaling, we used murine WT keratinocytes, PKP4-KO keratinocytes, and PKP4-KO keratinocytes overexpressing GFP-tagged PKP4 (Rescue cell line).

**Fig. 1:**
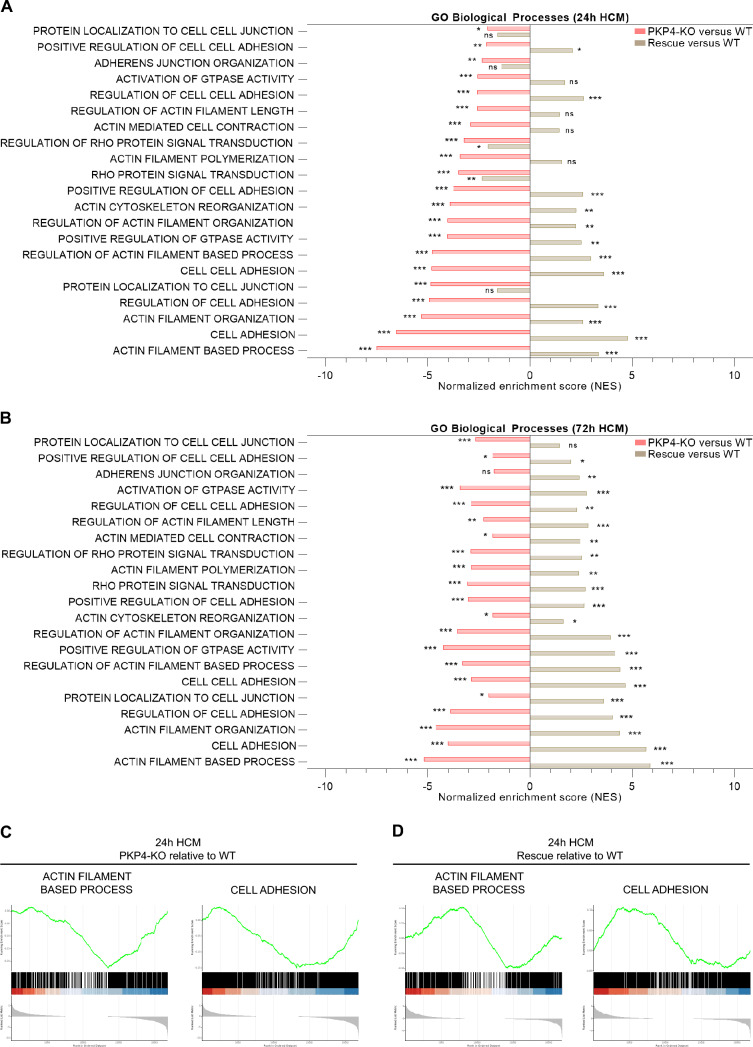
PKP4 regulates actin- and adhesion-associated pathway activities. **A**, **B** Normalized enrichment scores of selected gene sets among protein coding genes in PKP4-KO or Rescue (PKP4-KO + PKP4) versus WT cells after (**A**) 24 h HCM or (**B**) 72 h HCM. Positive values represent up regulation, negative values represent down regulation. **C**, **D** Enrichment plots for the two most significantly enriched Gene Ontology (GO) Biological Processes gene sets for **C** PKP4-KO relative to WT cells or (**D**) Rescue (PKP4-KO + PKP4) relative to WT cells after 24 h HCM incubation, respectively. The plots show the profile of the running enrichment scores and positions of gene set members on the rank-ordered list. Genes on the far left (red) correspond to the most upregulated actin- or adhesion-associated genes, whereas genes on the far right (blue) correspond to the most downregulated actin- or adhesion-associated genes. *P < 0.05; **P < 0.01; ***P < 0.001; ns, not significant. See also Fig. S1

Using these cells, we have performed gene expression analyses to identify molecular processes that are affected by PKP4. RNA sequencing of WT, PKP4-KO, and Rescue cells grown for 24 h or 72 h in LCM or HCM was performed followed by assessment of GSEA of protein coding genes to identify differentially regulated biological pathways and molecular functions. Signaling pathways associated with PKP4 were identified using the gene ontology databases [40]. The downregulated genes in PKP4-KO cells and the upregulated genes in rescue cells were primarily associated with actin- and adhesion-associated pathways. (Figs. 1A, B, S1A, B). In addition, GSEA enrichment plots show that PKP4 regulated genes involved in both, actin filament based processes and in cell adhesion (Figs. 1C, D, S1C, D). Taken together, these data indicate that the loss of PKP4 altered actin- and adhesion-associated pathway activities.

### PKP4 affects actin organization

**Fig. 2:**
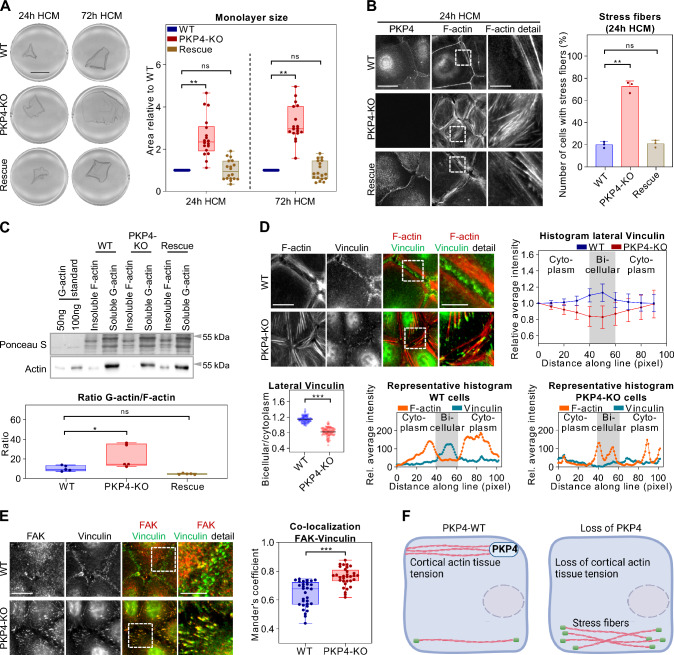
PKP4 affects actin organization. **A** Dispase-based tension assay of WT, PKP4-KO, and Rescue (PKP4-KO + PKP4) cells grown for 24 h or 72 h in HCM. Left: Representative images showing the monolayers detached from the culture flask. Scale bar = 5 mm. Right: Quantification of monolayer size relative to WT cells. Box plots show the monolayer area from eighteen independent experiments. The whiskers extend to the minimum and the maximum values. **B** Immunofluorescence analysis of F-actin organization. Left: Representative immunofluorescence images showing PKP4 and F-actin localization in WT, PKP4-KO, and Rescue (PKP4-KO + PKP4) cells grown for 24 h in HCM. Scale bar = 50 µm, detail 10 µm. Right: Number of cells with stress fibers. Averages + SD from three independent experiments are plotted. n ≥ 100 cells per condition. **C** G-actin/F-actin ratio in WT, PKP4-KO, and Rescue (PKP4-KO + PKP4) cells. Top: Representative western blot of actin. Ponceau S staining was used as a loading control. Bottom: Quantification of G-actin/F-actin ratio. Box plots show the fold change from five independent experiments. The whiskers extend to the minimum and the maximum values. **D** Immunofluorescence analysis of Vinculin and F-actin localization. Top: Representative immunofluorescence images showing F-actin and Vinculin localization in WT and PKP4-KO cells and a histogram of the relative average intensities of lateral Vinculin. Scale bar = 10 µm, detail 5 µm. Averages ± SD from n ≥ 100 cells per condition from two independent experiments are plotted. Bottom: Bicellular/cytoplasm ratio of Vinculin fluorescence intensity. n ≥ 100 cells per condition from two independent experiments are shown. Representative histograms of the relative average intensities of lateral F-actin and Vinculin in WT cells and PKP4-KO cells. **E** Immunofluorescence analysis of FAK and Vinculin localization in WT and PKP4-KO cells. Left: Representative immunofluorescence images showing FAK and Vinculin localization in WT and PKP4-KO cells. Scale bar = 10 µm, detail 5 µm. Right: Quantification of co-localization of FAK and Vinculin. Mander’s coefficient describes the amount of overlap in fluorescence intensity between two channels, here, the FAK and vinculin channel. It ranges from 0 for no co-localization to 1 for complete co-localization. Box plots show the Mander’s coefficient from n ≥ 30 images per condition from two independent experiments. The whiskers extend to the minimum and the maximum values. **F** Schematic depicting the difference in actin organization between WT and PKP4-KO cells. Created with biorender.com. *P < 0.05; **P < 0.01; ***P < 0.001; ns, not significant. Significance was determined by one-way ANOVA with Tukey’s multiple comparisons test (**A**–**C**) or by student’s unpaired two tailed t-test (**D**, **E**). See also Fig. S2

Since AJs and the associated actin cytoskeleton are major determinants of tissue tension and dynamics [41], we investigated the role of PKP4 in generating tension. Keratinocyte monolayers detached from the culture dish by dispase treatment typically contract due to intrinsic forces [42]. This contraction was essentially lost in PKP4-KO cells compared to their WT counterparts as determined by quantifying epithelial sheet areas (Fig. 2A). The effect was reversed in Rescue cells confirming a role of PKP4 in generating tissue tension.

Actin filaments and AJs function as tension sensors [9, 43]. Therefore, we asked if actin organization might be altered in PKP4-KO cells. WT cells and Rescue cells grown in HCM for 24 h to induce junction formation revealed a prominent cortical actin ring (Figs. 2B, S2A), whereas PKP4-KO cells displayed a striking increase in stress fibers (Fig. 2B). In order to find out if cortical ring formation was only delayed or completely inhibited, cells were grown for 120 h in HCM to allow junction maturation. Even after this prolonged incubation in differentiation medium, WT and Rescue keratinocytes showed low numbers of cells with stress fibers whereas PKP4-KO cells were characterized by a loss of the cortical actin ring and increased stress fibers (Fig. S2B), suggesting a general failure rather than a delay of cortical actin assembly.

Dynamic alterations of cellular organization depend on actin remodeling. During polymerization, actin undergoes a rapid transition from its globular, monomeric state (G-actin) to its filamentous (F-actin) form [44], revealing that the actin cytoskeleton is a highly dynamic structure. To analyze if the altered actin organization correlates with a change in the G-Actin to F-actin ratio, we determined the G-actin/F-actin ratios in WT, PKP4-KO, and Rescue cells (Fig. 2C). The loss of PKP4 resulted in an increased G-actin/F-actin ratio, suggesting that PKP4 promotes actin polymerization, which might facilitate cortical ring formation.

Stress fibers are often associated with focal adhesion contacts linking them to the underlying extracellular matrix [45]. Vinculin is a marker of focal adhesions but in addition has been recognized to localize at mature AJs that are under tension. Vinculin recruitment to AJs relies on force-dependent changes in α-catenin conformation [8, 46]. To analyze whether cytoskeletal changes affected vinculin distribution, WT and PKP4-KO cells were maintained in HCM for 24 h and processed for immunofluorescence (Fig. 2D). In WT keratinocytes, F-actin was particularly apparent at the cell periphery and vinculin localized predominantly at lateral AJs. In contrast, PKP4-KO cells lacked vinculin staining at the cell periphery. Here, vinculin localized at the tips of actin stress fibers suggesting that the loss of PKP4 led to vinculin localization at focal contacts. Signals from focal adhesions are transduced by the focal adhesion kinase (FAK) to regulate mechanosensing [47]. Co-staining of FAK and vinculin revealed increased co-localization of both proteins in PKP4-KO compared to WT cells (Fig. 2E). Since protein levels of FAK and vinculin were not affected by PKP4 (Fig. S2C), we assume that PKP4 primarily affected the localization of vinculin via generation of tension.

Taken together, we show that loss of PKP4 leads to extensive changes of the actin cytoskeleton, with an increase in cytoplasmic stress fibers at the expense of the cortical actin ring resulting in reduced tension (Fig. 2F).

### PKP4 modulates the morphology of junctions and stabilizes keratinocyte cell–cell adhesion

**Fig. 3:**
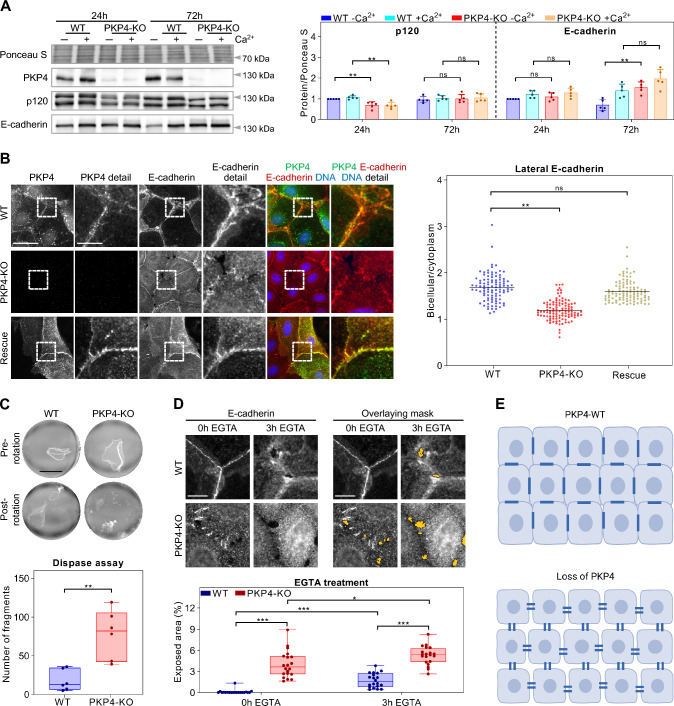
PKP4 modulates the morphology of junctions and stabilizes keratinocyte cell–cell adhesion. **A** Levels of AJ proteins in WT and PKP4-KO cells grown for 24 h or 72 h in medium with or without Ca^2+^. Left: Representative western blots of AJ proteins. Ponceau S staining was used as a loading control. Right: Quantification of protein amounts normalized to Ponceau S staining and relative to WT cells grown for 24 h in medium without Ca^2+^. Averages + SD from five independent experiments are plotted. **B** Immunofluorescence analysis of E-cadherin localization. Left: Representative immunofluorescence images showing PKP4 and E-cadherin localization in WT, PKP4-KO, and Rescue (PKP4-KO + PKP4) cells. Scale bar = 50 µm, detail 10 µm. Right: Bicellular/cytoplasm ratio of E-cadherin fluorescence intensity. n ≥ 100 cells per condition from two independent experiments. **C** Dispase-based dissociation assay of WT and PKP4-KO cells grown for 24 h or 72 h in HCM. Top: Representative images showing the results of dispase assays before and after mechanical stress. Scale bar = 5 mm. Bottom: Quantification of fragment numbers. Box plots show the fragment numbers from six independent experiments. The whiskers extend to the minimum and the maximum values. **D** Immunofluorescence analysis of E-cadherin localization in WT and PKP4-KO cells treated with EGTA (3.3 mM) for 3 h. Top left: Representative immunofluorescence images showing E-cadherin localization in WT and PKP4-KO cells before (0 h) or 3 h after EGTA treatment. Scale bar = 10 µm. Top right: Overlaying mask (yellow marked polygons) indicating exposed areas. Scale bar = 10 µm. Bottom: Quantification of the exposed areas. Box plots show the exposed area in percent per image from 20 images from two independent experiments. The whiskers extend to the minimum and the maximum values. **E** Schematic of junction morphology in WT compared to PKP4-KO cells. Created with biorender.com. *P < 0.05; **P < 0.01; ***P < 0.001; ns, not significant. Significance was determined by one-way ANOVA with Tukey’s multiple comparisons test (**A**, **B**) or by student’s unpaired two tailed t-test (**C**, **D**). See also Fig. S3

Actomyosin promotes cadherin clustering and thus AJ morphology. Based on the changes in actin organization and the localization of PKP4 in AJs we asked if AJ morphology and/or composition were altered in PKP4-KO keratinocytes. Cells were cultured in HCM for either 24 h to allow for junction formation or for 72 h to enable junction maturation and keratinocyte differentiation. Western Blot analysis revealed essentially unaltered expression of the AJ proteins analyzed (Fig. 3A). Unlike predicted, no compensatory upregulation of p120 was found. A transient and minor reduction of p120 was observed at 24 h after Ca^2+^ switch but the level was adjusted after 3 days in HCM.

To analyze whether the loss of PKP4 affects the morphology of AJs, WT, PKP4-KO, and Rescue keratinocytes were processed for immunofluorescence. AJ morphology was dramatically altered as revealed by E-cadherin staining (Fig. 3B). The loss of PKP4 resulted in an increased cytoplasmic pool of E-cadherin and decreased lateral E-cadherin localization with a punctate pattern instead of more continuous lines observed in WT and Rescue cells. Moreover, tricellular regions were not sealed. These findings indicate that loss of PKP4 interferes with AJ maturation and cortical actin organization.

Since AJs and desmosomes are interconnected [48], we asked whether PKP4 might alter the composition and morphology of desmosomes as well. Western blot analysis revealed a slight increase of differentiation-associated desmosomal proteins desmoglein 1 and PKP1 in PKP4-KO cells after 72 h HCM (Fig. S3A). The most profound effect was on desmoplakin 1/2, which was increased in PKP4-KO cells. Since desmoplakin 1/2 levels were considerably affected, WT, PKP4-KO, and Rescue keratinocytes were immunostained for desmoplakin (Fig. S3B). Similar to AJs, desmoplakin localization was altered with punctate instead of linear staining patterns in PKP4-KO cells. This suggests that the increased amounts of desmoplakin accumulate in the cytoplasm of PKP4-KO but did not incorporate into desmosomes. Again, the tricellular regions remained unsealed. To validate the dramatic loss of junctional proteins at tricellular regions in PKP4-KO cells, WT, PKP4-KO, and Rescue keratinocytes were immunostained for tricellulin, which accumulates primarily at tricellular junctions [49]. Tricellulin was decreased at tricellular regions in PKP4-KO cells (Fig. S3C), suggesting that PKP4 was required to close tricellular regions.

To analyze whether PKP4 interacts with desmosomal components, immunoprecipitation of endogenous PKP4 was performed. PKP1, PKP3, and desmoplakin did co-purify with PKP4 (Fig. S3D), suggesting that an association with PKP4 might facilitate desmosome assembly and/or alter desmosome composition.

To test whether PKP4 also affects formation of desmosomes, we performed a time course analysis of desmosome assembly in WT and PKP4-KO cells (Fig. S3E). HCM induced rapidly E-cadherin recruitment to lateral cell membranes in both, WT and PKP4-KO cells. The desmosomal markers desmoplakin and PKP3 also localized at lateral membranes after HCM treatment, but the loss of PKP4 delayed incorporation of desmoplakin and PKP3 into lateral junctions. These data suggest that PKP4 promotes desmosome formation.

Taken together, these data indicate that the loss of PKP4 results in a reduction of junctional proteins at lateral and tricellular membranes, suggesting that PKP4-KO cells fail to form mature junctions.

Since AJs and desmosomes maintain the mechanical integrity of cell–cell adhesion [50–53], we asked whether the PKP4-depdendent changes in morphology of AJs and desmosomes would correlate with changes in intercellular adhesion. Epithelial sheet assays were performed to test the strength of cell–cell adhesion (Fig. 3C). In line with the reduced association of junctional proteins with lateral and tricellular membranes in PKP4-KO cells, the loss of PKP4 reduced cell–cell adhesion as revealed by an increased number of fragments generated by mechanical stress. This confirms that PKP4 strengthens cell–cell adhesion.

Upon maturation, desmosomes become Ca^2+^-independent which marks a state of strong intercellular cohesion. To test if cell junctions in PKP4-KO cells would reach Ca^2+^-independence, WT and PKP4-KO cells were treated with EGTA and processed for immunofluorescence (Fig. 3D). In WT cells, EGTA treatment for 3 h induced small tricellular openings whereas lateral contacts remained intact as revealed by E-cadherin staining. In contrast, the loss of PKP4 resulted in a reduction of E-cadherin at tricellular contacts already in the absence of EGTA. Incubation with EGTA slightly increased tricellular gaps but in addition strongly reduced lateral E-cadherin staining. These data strongly support a role of PKP4 in junction maturation and stabilization.

Taken together, we propose that PKP4 promotes an association of AJ and desmosomal proteins with lateral and tricellular membranes to stabilize cell–cell adhesion. The loss of PKP4 resulted in decreased lateral localization of junctional proteins and increased tricellular gaps, which weakens cell–cell adhesion (Fig. 3E).

### PKP4 promotes ROCK-signaling

**Fig. 4:**
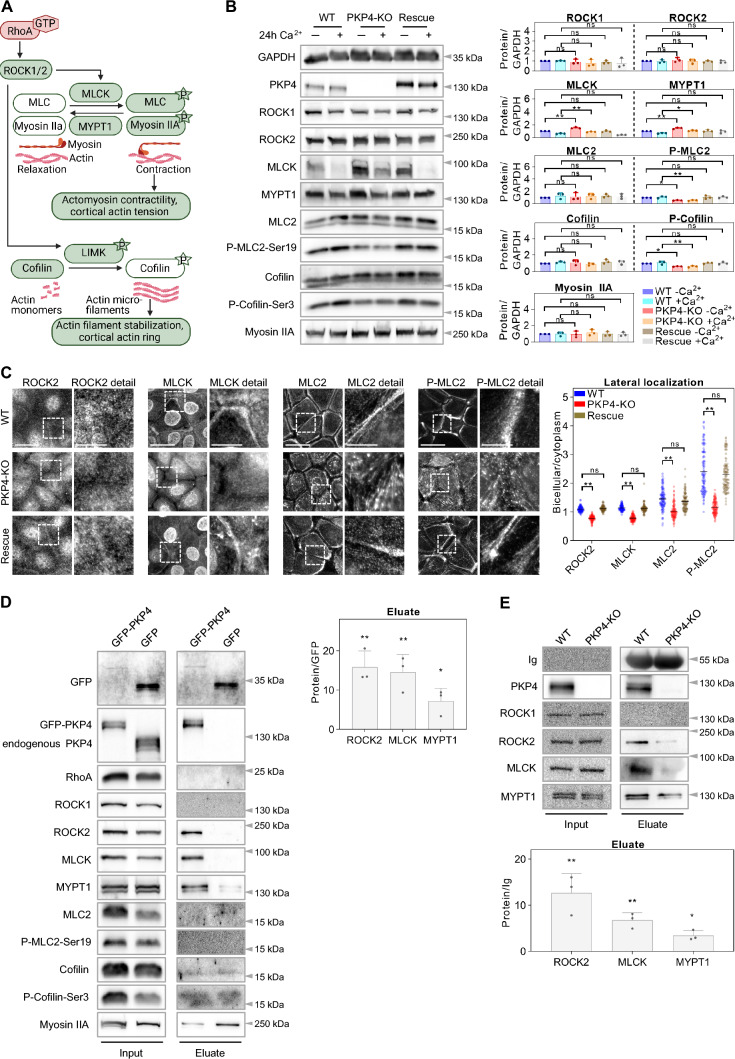
PKP4 promotes ROCK-signaling. **A** Schematic of ROCK-signaling. P – phosphorylation. Inactive proteins are shown in white. Created with biorender.com. **B** Amount and phosphorylation of proteins involved in ROCK-signaling. Left: Representative western blots of proteins in WT, PKP4-KO, and Rescue (PKP4-KO + PKP4) cells grown for 24 h in medium with or without Ca^2+^. GAPDH was used as a loading control. Right: Quantification of protein amounts normalized to GAPDH and relative to WT cells grown in medium without Ca^2+^. Averages + SD from three independent experiments are plotted. **C** Immunofluorescence analysis showing the localization of proteins involved in ROCK-signaling. Left: Representative immunofluorescence images showing ROCK2, MLCK, MLC2, and P-MLC2-Ser19 localization in WT, PKP4-KO, and Rescue (PKP4-KO + PKP4) cells. Scale bar = 50 µm, detail 10 µm. Right: Bicellular/cytoplasm ratio of fluorescence intensities. n ≥ 100 cells per condition from two independent experiments. **D** GFP-PKP4 or GFP was affinity-purified from WT + GFP or PKP4-KO + PKP4 cells. Left: Representative western blots of co-purified proteins. Right: Enrichment of ROCK2, MLCK, and MYPT1 normalized to precipitated GFP and relative to values of GFP cells (second lane in immunoblot, which was set to 1). Average + SD from three independent experiments was plotted. **E** Endogenous PKP4 was affinity-purified from WT cells. PKP4-KO cells were treated in parallel as control. Top: Representative western blots of input and co-purifying proteins. Bottom: Enrichment of ROCK2, MLCK, and MYPT1 normalized to Ig and relative to values of PKP4-KO cells (second lane in immunoblot, which was set to 1). Average + SD from three independent experiments was plotted. *P < 0.05; **P < 0.01; ***P < 0.001; ns, not significant. Significance was determined by one-way ANOVA with Tukey’s multiple comparisons test (**B**, **C**) or by student’s unpaired two tailed t-test (**D**, **E**). See also Fig. S4

Given that the expression of PKP4 is low compared to E-cadherin it seemed unlikely that PKP4 strengthens intercellular adhesion by directly recruiting E-cadherin and catenins. At the same time, intercellular adhesion increases with the application of force [54] supporting a role of actin organization in junction stability. Therefore, we hypothesized that the effects of PKP4 depletion on junctions may be mediated by the loss of cortical actin organization and thus focused on regulators of actin polymerization and actomyosin tension. The dynamic organization of the actin cytoskeleton is regulated by Rho GTPases [55]. GTP-bound RhoA activates ROCK1/2 which leads to activation of myosin light chain kinase (MLCK) and inhibition of MLC phosphatase (MYPT1), resulting in an increase of MLC phosphorylation (P-MLC) to induce actomyosin-based contractility [56]. ROCK also directly phosphorylates LIMK, which results in downstream phosphorylation and inactivation of the actin depolymerizing factor cofilin leading to filament stabilization [57] (Fig. 4A).

To analyze the putative effect of PKP4 on ROCK-signaling, we quantified the amounts and activation of several ROCK1/2 effectors in WT, PKP4-KO, and Rescue cells maintained in LCM or HCM for 24 h by western blotting (Fig. 4B). Protein levels of ROCK1, ROCK2, MLC2, cofilin, and myosin IIA were unaltered by PKP4. More importantly, the loss of PKP4 led to increased protein levels of the opponents MLCK and MYPT1 suggesting increased actin dynamics. Strongly decreased phosphorylation of MLC2 supports the reduced contractility observed in the PKP4-KO keratinocyte sheets (Fig. 2A). Furthermore, decreased Cofilin phosphorylation in PKP4-KO cells fosters the data showing increased G-actin levels in these cells (Fig. 2C).

Since cortical actin was essentially lost but stress fibers were increased in PKP4-KO cells, we wondered if this correlates with altered localization of ROCK1/2 and their effectors. WT, PKP4-KO and Rescue keratinocytes were maintained for 24 h in HCM and processed for immunofluorescence. Whereas localization of ROCK1, MYPT1, cofilin, phospho-cofilin, and myosin IIA was unaltered in PKP4-KO cells (Fig. S4), lateral localization of ROCK2 and MLCK was reduced in PKP4-KO cells compared to WT and Rescue cells (Fig. 4C). Moreover, PKP4 dramatically promoted the lateral localization of total MLC2 and phospho-MLC2.

To analyze whether PKP4 interacts with effectors of ROCK1/2-signaling to modulate actin dynamics, GFP-PKP4 was affinity purified from Rescue cells (Fig. 4D). RhoA, ROCK1, MLC2, phospho-MLC2, as well as cofilin, phospho-cofilin, and myosin IIA, did not co-purify with PKP4. In contrast, ROCK2, MLCK, and MYPT1 co-precipitated. This was further validated in an immunoprecipitation of endogenous PKP4 which revealed again a co-precipitation of ROCK2, MLCK, and MYPT1 in WT cells (Fig. 4E), suggesting that an association with PKP4 might alter their activity to control actin dynamics at the cortical ring.

Taken together we show that PKP4 promotes MLC and cofilin phosphorylation to regulate actomyosin-dependent tension and cortical ring formation. PKP4 dramatically increased the lateral localization of active phospho-MLC2, which facilitates actomyosin contraction at the cortical ring. These data suggest disturbed mechanosignaling in PKP4-KO keratinocytes. We conclude that PKP4 might function as a scaffold to locally regulate a ROCK2-MLCK-MLC2 axis to promote cortical actin tension.

### PKP4 promotes RhoA activation at the cell cortex and cortical actin ring formation

The balance of the small GTPases RhoA, Rac1, and Cdc42 controls actin cytoskeleton dynamics through ROCK signaling [58]. The disruption of RhoA activity reduced phosphorylation of MLC2 [59] as observed in PKP4-KO cells. To analyze if RhoA activity would be affected, a RhoA activation assay was performed to measure total RhoA activities (Fig. S5A). In accordance with decreased phosphorylation of MLC2, the loss of PKP4 reduced total RhoA activity.

**Fig. 5:**
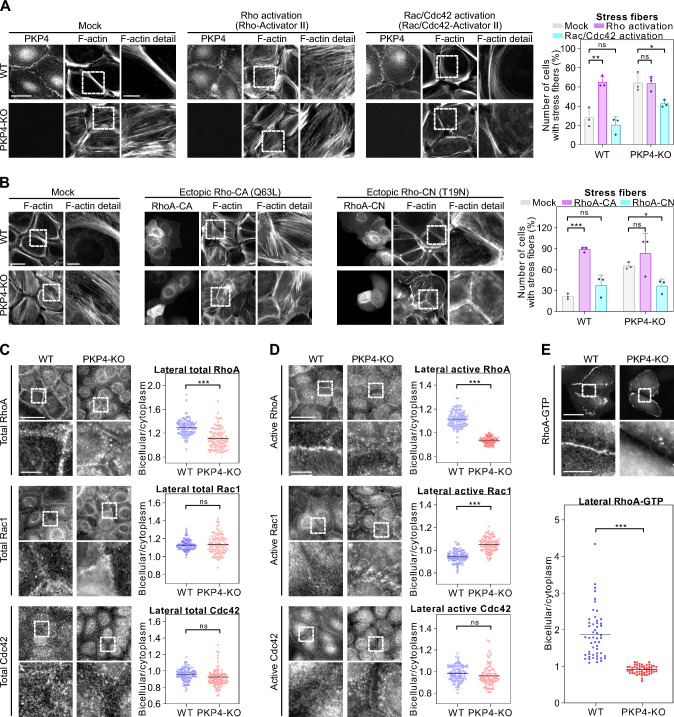
PKP4 promotes RhoA activation at the cell cortex and cortical actin ring formation. **A** Immunofluorescence analysis of F-actin organization after Rho or Rac/Cdc42 activation. Left: Representative immunofluorescence images showing PKP4 and F-actin localization in WT and PKP4-KO cells treated with PBS (Mock), Rho activator II (5 µg/ml), or Rac/Cdc42 activator II (5 units/ml). Scale bar = 50 µm, detail 10 µm. Right: Number of cells with stress fibers. Averages + SD from three independent experiments are plotted. n = 50 cells per condition. **B** Immunofluorescence analysis of F-actin organization in constitutive active or negative RhoA expressing cells. Left: Representative immunofluorescence images showing F-actin localization in WT and PKP4-KO cells after ectopic expression of constitutive active RhoA (RhoA-CA(Q63L)) or constitutive negative RhoA (RhoA-CN(T19N)). Scale bar = 50 µm, detail 10 µm. Right: Number of cells with stress fibers. Averages + SD from three independent experiments are plotted. n = 10 cells per condition. **C** Immunofluorescence analysis showing total RhoA, Rac1, and Cdc42 localization. Left: Representative immunofluorescence images showing total RhoA, Rac1, or Cdc42 localization in WT and PKP4-KO cells. Scale bar = 100 µm, detail 10 µm. Right: Bicellular/cytoplasm ratio of fluorescence intensity. n ≥ 100 cells per condition from two independent experiments. **D** Immunofluorescence analysis of the localization of active RhoA, Rac1, and Cdc42. Left: Representative immunofluorescence images showing active RhoA, Rac1, or Cdc42 localization in WT and PKP4-KO cells. Scale bar = 100 µm, detail 10 µm. Right: Bicellular/cytoplasm ratio of fluorescence intensity. n ≥ 100 cells per condition from two independent experiments. **E** Immunofluorescence analysis showing the localization of a RhoA-GTP biosensor. Top: Representative immunofluorescence images showing EGFP-RhoA biosensor localization in WT and PKP4-KO cells. Scale bar = 50 µm, detail 10 µm. Bottom: Bicellular/cytoplasm ratio of fluorescence intensity. n ≥ 50 cells per condition from two independent experiments. *P < 0.05; **P < 0.01; ***P < 0.001; ns, not significant. Significance was determined by student’s unpaired two tailed t-test (A, B, C, D, E). See also Fig. S5

To analyze the roles of the Rho-GTPases Rho, Rac, and Cdc42 in stress fiber and cortical actin organization in more detail, WT, PKP4-KO, and Rescue cells were treated with Rho or Rac/Cdc42 activators and processed for immunofluorescence (Fig. 5A). Rho activation induced stress fiber formation in WT cells whereas Rac/Cdc42 activation suppressed stress fibers in PKP4-KO cells. To further validate the PKP4-dependent regulation of Rho activity, constitutively active or constitutively negative mutants of RhoA GTPases were ectopically expressed in WT and PKP4-KO cells, followed by immunofluorescence (Fig. 5B). In agreement with the Rho activator studies, constitutively active RhoA increased stress fiber formation in WT cells whereas constitutively negative RhoA suppressed stress fibers in PKP4-KO cells suggesting that PKP4 affects the activity of Rho-GTPases.

To resolve the apparent contradiction between the reduced total RhoA activity found in PKP4-KO cells and the fact that hyperactive RhoA mimicked the PKP4-phenotype, we focused on the local regulation of Rho-GTPases. Active RhoA was shown to localize in the cytoplasm and at the membrane but to activate effector proteins primarily when localized at the membrane where RhoA needs to be stabilized to engage downstream pathways of contractility [60]. Therefore, we asked if PKP4 would contribute to the stabilization of active RhoA at the membrane. To evaluate if the cortical localization and/or activity of RhoA, Rac1, and/or Cdc42 was indeed dependent on PKP4, WT and PKP4-KO cells were processed for immunofluorescence (Fig. 5C, D). The loss of PKP4 correlated with decreased lateral localization of both total and active RhoA. Rac and Cdc42 localization were essentially unaffected but lateral localization of active Rac1 was increased in PKP4-KO cells compared to WT cells whereas Cdc42 activity was unaltered. These data suggest that PKP4 promotes the localization of active RhoA at the lateral membrane but suppresses lateral Rac1 activity.

To further validate a PKP4-dependent localization of active RhoA, a RhoA biosensor was ectopically expressed in WT and PKP4-KO cells and the cells were processed for immunofluorescence (Figs. 5E, S5B). This sensor visualizes endogenous Rho-GTP. Like E-cadherin, the sensor accumulated strongly at the lateral membranes in WT cells, indicating that RhoA was locally active at the plasma membrane. In contrast, the loss of PKP4 resulted in the loss of lateral RhoA-GTP which instead showed a cytoplasmic localization although E-cadherin membrane association was unaltered. This indicates reduced RhoA activity at the lateral membrane of PKP4-KO cells.

Taken together, we show that PKP4 suppresses cytoplasmic RhoA activation to restrain stress fiber formation but promotes RhoA activity at lateral membranes to facilitate cortical actin ring formation and the generation of tension through a RhoA-ROCK-MLCK-MLC2 axis.

### PKP4-dependent actin dynamics and tension are regulated by ARHGAP23 and ARHGEF2

Local changes in Rho-GTPase activity in the PKP4-KO cells raised the question how PKP4 modulates Rho-signaling. Spatio-temporal control of Rho-GTPases depends on the local balance of GEF and GAP activities. Therefore, we hypothesized that PKP4 might affect Rho-signaling by modulating GEF and/or GAP localization and/or activities at keratinocyte AJs.

**Fig. 6:**
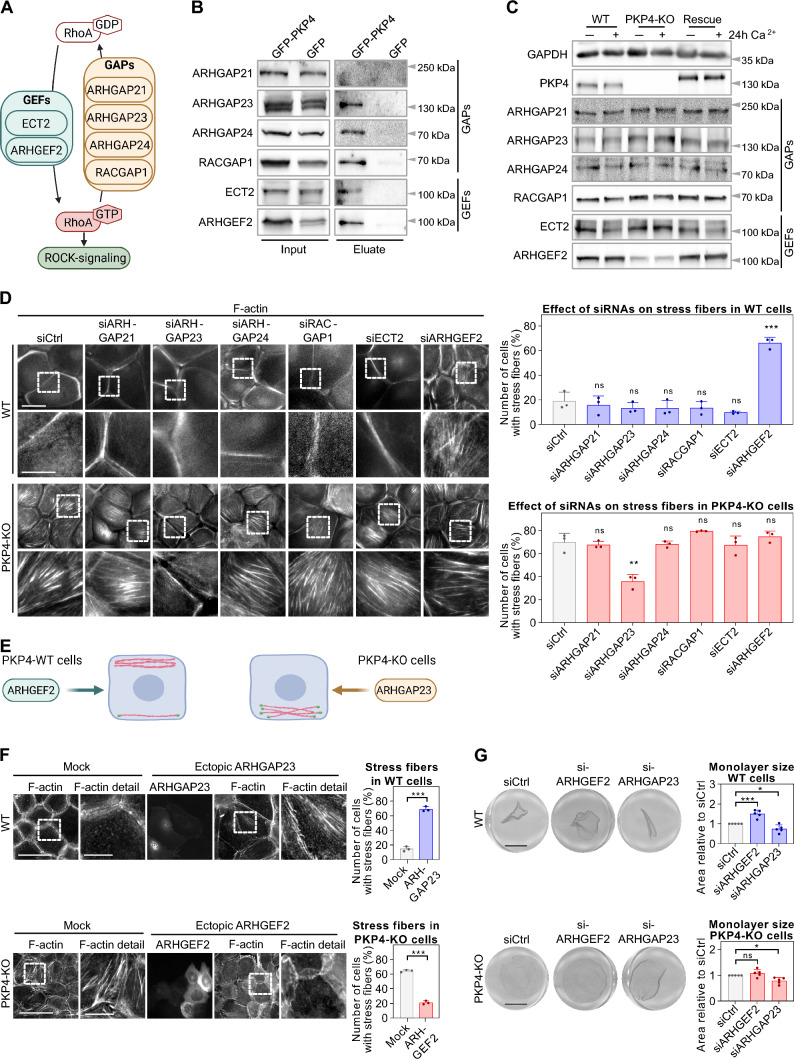
PKP4-dependent actin dynamics and tension are regulated by ARHGAP23 and ARHGEF2**. A** Schematic of selected GEFs and GAPs and their role in promoting or inhibiting RhoA-activity, respectively. Created with biorender.com. **B** GFP-PKP4 or GFP was affinity-purified from WT + GFP or PKP4-KO + PKP4 cells. Representative western blots of co-purified proteins. **C** Protein level of selected GEFs and GAPs. Left: Representative western blots of GEF and GAP proteins in WT, PKP4-KO, and Rescue (PKP4-KO + PKP4) cells grown for 24 h in medium with or without Ca^2+^. GAPDH was used as a loading control. **D** Immunofluorescence analysis of the F-actin organization in siRNA treated WT and PKP4-KO cells. Left: Representative immunofluorescence images showing F-actin localization in WT and PKP4-KO cells after knockdown of the indicated GEFs and GAPs. Scale bar = 50 µm, detail 10 µm. Right: Number of cells with stress fibers. Averages + SD from three independent experiments are plotted. n ≥ 100 cells per condition. **E** Schematic of the effects of ARHGEF2 and ARHGAP23 in WT and PKP4-KO cells. Created with biorender.com. **F** Immunofluorescence analysis of F-actin organization in WT cells ectopically expressing ARHGAP23 and in PKP4-KO cells ectopically expressing ARHGEF2. Left: Representative immunofluorescence images showing F-actin localization in WT and PKP4-KO cells after ectopic expression of ARHGAP23 or ARHGEF2, respectively. Scale bar = 100 µm, detail 10 µm. Right: Number of cells with stress fibers. Averages + SD from three independent experiments are plotted. n ≥ 30 cells per condition. **G** Dispase-based tension assay of WT and PKP4-KO cells treated with ARHGEF2- or ARHGAP23-directed siRNAs. Left: Representative images showing the monolayers. Scale bar = 5 mm. Right: Quantification of monolayer size relative to non-targeting siRNA (siCtrl) treated cells. Averages + SD from five independent experiments are plotted. *P < 0.05; **P < 0.01; ***P < 0.001; ns, not significant. Significance was determined by student’s unpaired two tailed t-test (D, F, G). See also Fig. S6

GEFs promote the release of GDP in exchange for GTP to activate the GTPase. In contrast, GAPs increase the intrinsic hydrolytic activity that converts GTP into GDP thereby inactivating the GTPase [10, 11]. In order to find out how PKP4 modulates the activity of Rho-GTPases we investigated interactions with these upstream regulators. For this purpose, specific GEFs and GAPs were selected for a detailed characterization based on several criteria (Fig. 6A): First, we analyzed those candidates that were already known to associate with PKP4 in a different context. A direct interaction between PKP4, the GEF ECT2, and RhoA has been previously identified during cytokinesis [20]. Moreover, RACGAP1 [61] and ARHGAP21 had been described as a PKP4 interacting protein [12]. Since ARHGAP23 is similar to ARHGAP21 and both contain a PDZ domain, which might mediate PKP4 binding via its PDZ binding motif, ARHGAP23 was also included. Secondly, GEFs and GAPs that were known to modulate cell junctions were examined. ARHGEF2 regulates the assembly of AJs [62], and ARHGAP24 promotes the formation of AJs by accumulating E-cadherin [63].

To analyze whether PKP4 interacts with selected GEFs and GAPs, PKP4-GFP was affinity-purified from Rescue cells (Fig. 6B, for quantification see Fig. S6A). ARHGAP21 did not co-purify with PKP4. In contrast, the GAPs ARHGAP23, ARHGAP24, and RACGAP1 co-precipitated. In addition, the GEFs ECT2 and ARHGEF2 (also called GEFH1) were both co-precipitated. The co-precipitation indicated an association with PKP4 and emphasized a role of PKP4 as a scaffold in the spatio-temporal control of Rho signaling.

The function of GEFs and GAPs is regulated by expression as well as localization. To analyze a putative effect of PKP4 on the protein level of the selected GEFs and GAPs, we quantified their amounts in WT, PKP4-KO, and Rescue cells maintained for 24 h in LCM where PKP4 is cytoplasmic or in HCM where PKP4 localizes at cell junctions (Fig. 6C, for quantification see Fig. S6B). Protein levels of ARHGAP21, ARHGAP24, RACGAP1, and ECT2 were unaltered by PKP4 as determined by western blotting. More importantly, the loss of PKP4 slightly increased ARHGAP23 protein expression but dramatically decreased the ARHGEF2 protein level. These effects were reversed in Rescue cells, supporting a role of PKP4 in regulating ARHGAP23 and ARHGEF2 levels.

To directly link the selected GEFs and GAPs with actin dynamics, the impact of siRNA-mediated repression of these GEFs and GAPs was studied with respect to actin organization. WT and PKP4-KO cells were treated with control (siCtrl) or GEF/GAP-directed siRNAs (siARHGAP21, siARHGAP23, siARHGAP24, siRACGAP1, siECT2, siARHGEF2), maintained for 24 h in HCM, and processed for immunofluorescence (Fig. 6D, for quantification of knockdown efficiencies see Fig. S6C). ARHGEF2 depletion increased stress fibers in WT cells whereas control-treated WT cells revealed a cortical actin ring, suggesting that ARHGEF2 depletion reduced cortical RhoA activity and thus mimicked the effect of PKP4 loss. In PKP4-KO cells, ARHGAP23 depletion correlated with a loss of stress fibers, suggesting that ARHGAP23 is active in the PKP4-KO cell cytoplasm to reduce cortical RhoA activity and suppress cortical ring formation. Thus, ARHGEF2 and ARHGAP23 might be the key regulators for actin organization in a PKP4-dependent manner (Fig. 6E).

Based on these findings, we predicted that overexpression of ARHGAP23 should promote stress fibers in WT cells. Transfected cells were maintained for 24 h in HCM and processed for immunofluorescence (Fig. 6F). In accordance with our assumption, overexpressed ARHGAP23 partially localized at the cell cortex where it could inactivate RhoA to prevent cortical actin ring formation and led to increased stress fibers in WT cells. Moreover, we wondered if overexpression of ARHGEF2 in PKP4-KO cells might partially rescue cortical actin ring formation because of a general massive increase in its activity although PKP4 would not mediate its cortical localization. Indeed, overexpression of ARHGEF2 reduced stress fibers in PKP4-KO and improved cortical actin to some extent.

To reveal the functional consequences of the PKP4-dependent actin regulation by ARHGAP23 and ARHGEF2 we investigated if this would also affect cell tension. WT and PKP4-KO cells maintained for 24 h in HCM were treated with control (siCtrl), ARHGEF2-directed (siARHGEF2), or ARHGAP23-directed (siARHGAP23) siRNAs and processed for the dispase assay (Fig. 6G). ARHGEF2 depletion in WT cells increased the sheet area, indicative of reduced mechanical tension in accordance with reduced cortical actin and increased stress fibers observed in these cells. In PKP4-KO cells, depletion of ARHGEF2 did not affect sheet area probably because ARHGEF2 levels were already severely downregulated by the loss of PKP4. In contrast, ARHGAP23 depletion in PKP4-KO cells significantly reduced the sheet area, suggesting that the elevated ARHGAP23 level is connected to reduced mechanical tension. Although the ARHGAP23 level was lower in WT than in PKP4-KO cells, depletion of ARHGAP23 further reduced the sheet area of WT cells confirming that ARHGAP23 negatively controls tension. In addition to the effects on cell tension, the regulation of RhoA also affects keratinocyte adhesion (Fig. S6D) where ARHGEF2 depletion in WT cells interfered with stable cell–cell adhesion as indicated by increased numbers of fragments generated by mechanical stress. In contrast, ARHGEF2 depletion had no significant effect on intercellular cohesion in PKP4-KO cells, where the ARHGEF2 level was already strongly reduced. ARHGAP23 depletion decreased the number of fragments in PKP4-KO and WT cells, suggesting increased cell–cell adhesion.

Taken together, our data reveal that PKP4 affects actin organization through ARHGAP23 and ARHGEF2. PKP4 promotes ARHGEF2 expression but suppresses ARHGAP23 levels resulting in cortical ring formation and increased mechanical tension. By interacting with both proteins, PKP4 could control their localization and balance their activities.

### Junctional localization of ARHGAP23 and ARHGEF2 is regulated by PKP4

**Fig. 7:**
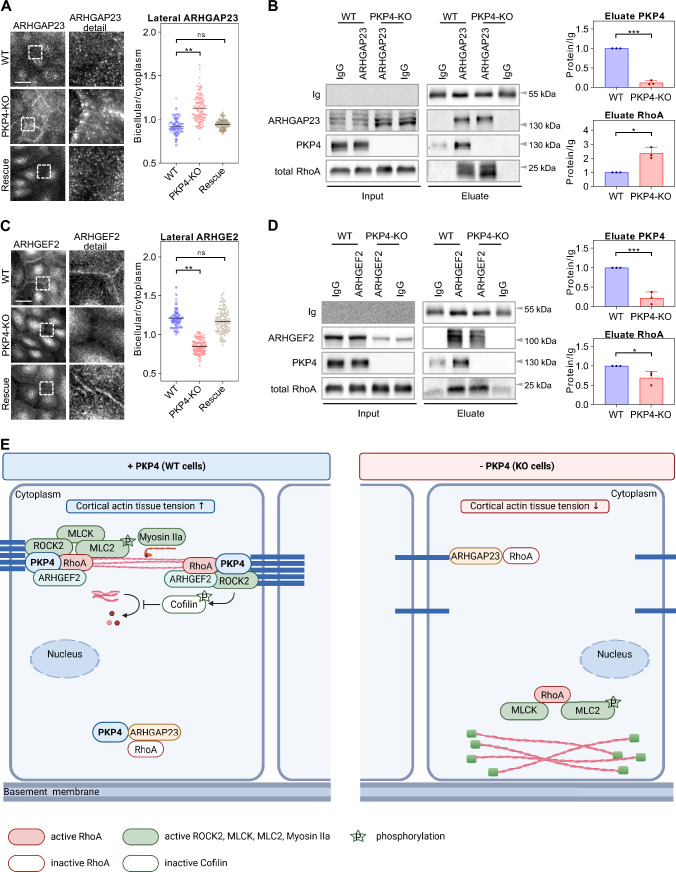
Junctional localization of ARHGAP23 and ARHGEF2 is regulated by PKP4. **A** Immunofluorescence analysis of the junctional localization of ARHGAP23. Left: Representative immunofluorescence images showing ARHGAP23 localization in WT, PKP4-KO, and Rescue (PKP4-KO + PKP4) cells. Scale bar = 50 µm, detail 10 µm. Right: Bicellular/cytoplasm ratio of fluorescence intensity. n ≥ 100 cells per condition from two independent experiments. **B** Endogenous ARHGAP23 was affinity-purified from WT and PKP4-KO cells. Left: Representative western blots of co-purifying proteins. Normal rabbit IgG served as negative control. Right: Quantification of eluate protein levels normalized to IgG heavy chain and relative to values in control cells. Averages + SD from three independent experiments are plotted. **C** Immunofluorescence analysis of the junctional localization of ARHGEF2. Left: Representative immunofluorescence images showing ARHGEF2 localization in WT, PKP4-KO, and Rescue (PKP4-KO + PKP4) cells. Scale bar = 50 µm, detail 10 µm. Right: Bicellular/cytoplasm ratio of fluorescence intensity. n ≥ 100 cells per condition from two independent experiments. **D** Endogenous ARHGEF2 was affinity-purified from WT and PKP4-KO cells. Left: Representative western blots of co-purifying proteins. Normal rabbit IgG served as negative control. Right: Quantification of eluate protein levels normalized to IgG heavy chain and relative to values in control cells. Averages + SD from three independent experiments are plotted. **E** Proposed model of PKP4’s role in Rho signaling. Created with biorender.com. *P < 0.05; **P < 0.01; ***P < 0.001; ns, not significant. Significance was determined by one-way ANOVA with Tukey’s multiple comparisons test (**A**, **C**) or by student’s unpaired two tailed *t*-test (**B**, **D**)

Since GEFs and GAPs are essential for the local control of RhoA activity [64], we expected that PKP4 might direct the localization of ARHGAP23 and ARHGEF2 to control their function. To analyze a putative junctional localization of ARHGAP23, WT, PKP4-KO, and Rescue cells maintained for 24 h in HCM were processed for immunofluorescence (Fig. 7A). ARHGAP23 was not only upregulated in PKP4-KO cells, but also showed increased lateral localization compared to WT and Rescue cells, suggesting that PKP4 displaced ARHGAP23 from junctions. To test whether the mislocalized ARHGAP23 in PKP4-KO cells still interacted with RhoA, endogenous ARHGAP23 was affinity purified from WT and PKP4-KO cells maintained for 24 h in HCM and probed for co-purifying proteins by western blotting with the indicated antibodies (Fig. 7B). PKP4 and total RhoA were co-precipitated by ARHGAP23 from WT cells confirming an association as shown in Fig. 6B. Importantly, the amount of total RhoA co-precipitating with ARHGAP23 was elevated in PKP4-KO compared to WT cells. Thus, we conclude that an increased ARHGAP23-RhoA interaction in PKP4-KO cells might diminish local RhoA activity at junctions and the cell cortex and promote stress fibers.

Finally, we investigated the junctional localization of ARHGEF2 in WT, PKP4-KO, and Rescue cells maintained for 24 h in HCM and processed for immunofluorescence (Fig. 7C). Loss of PKP4 interfered with the lateral ARHGEF2 localization. Taken together with the reduction in ARHGEF2 protein level, this suggests that PKP4 recruits ARHGEF2 to AJ and stabilizes cortical ARHGEF2 by association. To probe for RhoA association, endogenous ARHGEF2 was affinity purified from WT and PKP4-KO cells maintained for 24 h in HCM and probed by western blotting with the indicated antibodies (Fig. 7D). PKP4 and total RhoA were co-precipitated, confirming the association between ARHGEF2 and PKP4. The association with RhoA was retained in PKP4-KO cells although the amount of the complex was reduced due to reduced ARHGEF2 levels. Thus the reduced level of ARHGEF2 in PKP4-KO cells did not interfere with an interaction with RhoA in general but the altered localization of ARHGEF2 shifted RhoA activity from cell adhesions to the cytoplasm thereby preventing cortical ring formation but supporting stress fibers.

Collectively, we have demonstrated that PKP4 acts as a local scaffold that regulates the generation of intrinsic forces by promoting ARHGEF2 and preventing ARHGAP23 localization at cell borders resulting in local RhoA activation and reorganizing of actin and AJs.

## Discussion

AJ are dynamic structures that connect epithelial cells to form coherent cell sheets. Several studies have identified cellular contractility as an important determinant of junctional function [65]. Contractile tension at AJs is generated by an actomyosin cortex. Its formation depends on the recruitment of signaling proteins to AJ including RhoA, which activates non-muscle myosin [22, 66]. In addition, a conversion of disorganized actin filament networks into bundles is required for AJ maturation with formation of a zonula adherens (ZA) [67].

Here we have identified a role of the AJ associated protein PKP4 in the generation of tension across keratinocyte epithelial sheets. We show that the generation of intrinsic force requires a PKP4-dependent regulation of RhoA activity. We propose that PKP4 provides a scaffold for the RhoA activator ARHGEF2 at AJs which allows the recruitment of RhoA and its effectors MLCK and MLC to AJs to facilitate cortical ring formation and actomyosin contraction. In contrast, PKP4 association with ARHGAP23 reduced ARHGAP23 binding to RhoA and this occurred primarily in the cytoplasm thereby reducing stress fiber formation (summarized in Fig. 7E).

### The AJ proteins PKP4, p120, and α-catenin differentially regulate Rho-signaling

Among the E-cadherin associated cytoplasmic plaque proteins of AJs, α-catenin and p120 are known to be involved in the spatiotemporal control of Rho signaling: The centralspindlin complex, which consists of KIF23 (MKLP1) and RacGAP1 (MgcRacGAP), is known to control contractile ring formation and cell abscission during cytokinesis [68]. The same complex was shown to localize at the ZA in interphase cells by interacting with α-catenin. α-catenin recruited ECT2 to activate RhoA and supported junctional integrity through tension generated by myosin IIA. Moreover, centralspindlin inhibited the junctional localization of ARHGAP5 thereby preventing RhoA inactivation [22]. α-catenin dependent RhoA activation increased actomyosin contractility at AJs leading to tension induced conformational changes in α-catenin. This α-catenin activation is a key event in the dynamic regulation of AJ remodeling and allows recruitment of specific actin-binding proteins, including vinculin. Thus, α-catenin regulates not only force sensing, but also force transmission, through a Rho-mediated feedback mechanism [8].

In contrast, p120 potently inhibited RhoA activity and in addition activated Rac and Cdc42 [69, 70]. p120 suppressed the intrinsic GDP/GTP exchange activity of RhoA in a manner comparable to that of guanine nucleotide dissociation inhibitors (GDI) [13]. By directly binding to RhoA, p120 sequestered RhoA in an inactive state. p120 also regulated actomyosin contractility by controlling RhoA GTPase cycling at the centralspindlin complex during cytokinesis. RhoA activity was mislocalized in anaphase and telophase in p120 depleted cells resulting in cytokinesis defects [71].

PKP4 was shown to regulate cytokinesis and cell division by regulating Rho activity [19, 20]. This required a direct interaction with RhoA and the Rho-GEF Ect2. However, a role of PKP4 in junction associated Rho-signaling has remained elusive so far. In keratinocytes, the activation of RhoA was necessary for the formation and stability of AJs, as RhoA inhibition resulted in E-cadherin removal from the junctional complex, and actomyosin contractility was crucial for confluence-dependent inhibition of keratinocyte proliferation [72].

Here we show that in PKP4-KO keratinocytes, actin organization and Rho-GTPase activity are profoundly altered, raising the question how PKP4 modulates Rho-signaling beyond cytokinesis. Since the spatio-temporal control of Rho-GTPases depends on the local balance between GEF and GAP activities, we hypothesized that PKP4 might affect Rho-signaling by interacting with GEFs and/or GAPs at the AJs. We identified ARHGEF2 and ARHGAP23 as highly affected by PKP4. Whereas ARHGEF2 was recruited to AJs in a PKP4-dependent manner, ARHGAP23 was rather displaced from the cell cortex suggesting that PKP4 interfered with its junction-associated activity. The local activity of ARHGEF2 together with the displacement of ARHGAP23 can both contribute to enhanced RhoA activity at the ZA which through the activation of a ROCK-MLCK-MLC signaling axis enables actomyosin contractility and the generation of tension.

These data imply Rho-signaling as a general feature of the p120 family of proteins. However, our results demonstrate that p120 and PKP4 exert opposing roles in Rho-signaling. What does this mean in the context of the epidermis? Since PKP4 expression is restricted to the basal layers [73] its function in Rho signaling appears important for actively proliferating cells but dispensable for differentiated cells. A main function of the suprabasal cells is to provide a stable barrier. In contrast, epidermal renewal depends on dynamic reorganization of the cytoskeleton and cell shape and requires cell extrusion from the basal layer. During physiological self-renewal, proliferation within the basal layer leads to crowding, thereby locally distorting cell shape and stress distribution resulting in a decrease in cortical tension. This triggered differentiation and delamination of basal cells, reinstating basal cell layer density [74]. Epidermal renewal is balanced with differentiation and the movement of stem cells to the barrier-forming layers of the epidermis. According to our data, PKP4 would prevent the loss of tension and thus differentiation in most basal cells through the activation of RhoA and actomyosin at the cell cortex. In contrast, in cells committed to differentiation, PKP4 is down-regulated leading to reduced tension thus facilitating delamination. In accordance to this hypothesis, Spindler et al. [75] have shown that RhoA signaling is primarily important in the lower epidermis to maintain adhesion of keratinocytes, whereas in superficial layers, the role of RhoA signaling seemed to be less pronounced. Additionally, p120 could counteract the function of PKP4 to guarantee the balance of forces and maintain barrier function in the suprabasal layers. In support of this notion, a conditional knockout of p120 in the epidermis caused decreased epidermal differentiation and interfered with barrier function [76].

### A role of PKP4 in the pathogenesis of skin diseases

Altered cellular forces, AJs, and RhoA signaling have all been implicated in cancer. RhoA mutations appear restricted to certain cancer types and distinct RhoA mutational hotspots are associated with different cancer types [77, 78]. However, mutations in RhoA are not frequent and accumulating evidence supports a role for its regulators including ARHGEF2 as well as the effectors ROCK1/2 and MLCK in tumor development and progression. Genetic analyses uncovered a strong correlation between the level of ARHGEF2 expression and cancer progression and the development of drug resistance. ARHGEF2 transcription was increased by oncogenic *p53* mutations [79] and the activation of RhoA appeared to be intrinsic to its oncogenic abilities. ROCK-driven actomyosin contractility controls a series of processes including cell morphology, cell migration, invasion, differentiation, proliferation, and resistance to apoptosis. Accordingly, Rho-ROCK-myosinII signaling was shown to play a role not only in primary tumor formation but also in cell migration, dissemination, and metastasis. Therefore, numerous ROCK inhibitors have been investigated for their therapeutic potential in the treatment of cancers [80]. Abnormal expression of MLCK has been observed in many diseases including cancer where MLCK-dependent phosphorylation of myosin II increased the metastatic potential of tumor cells [81]. Based on our findings that PKP4 is an important regulator of an ARHGEF2-RhoA-ROCK/MLCK signaling axis, it is tempting to speculate that this function of PKP4 may contribute to cancer. In support of this notion, PKP4 has been implicated in pancreatic adenocarcinoma and thymoma (http://gepia2.cancer-pku.cn, [82]).

Beyond its potential role in cancer, distorted Rho signaling has also been implicated in the autoimmune diseases Pemphigus Foliaceus (PF) and Pemphigus vulgaris. Most notably, PF-antibodies induced skin blistering by interfering with RhoA signaling which compromised intercellular adhesion and ultimately induced keratinocyte dissociation [83]. In a variant of endemic PF, autoantibodies directed against PKP4 and the related armadillo repeat gene deleted in velo-cardio-facial syndrome (ARVCF) protein were detected, pointing to a potential role of PKP4 in the pathomechanism of Pemphigus skin diseases.

## Conclusions and limitations

Intrinsically generated forces at the junctions serve to balance cell proliferation and differentiation during skin renewal. Our data suggest that PKP4 plays a role in this context by dynamically regulating the spatio-temporal activity of Rho signaling. This raises several questions that require further investigation: First, the mechanism how PKP4 regulates the levels of the RhoA regulators ARHGEF2 and ARHGAP23 has currently not been resolved. So far, no data are available to link PKP4 with transcriptional or post-transcriptional regulation. Second, we have tested the response of murine keratinocytes to PKP4-mediated cell tension. However, our study did not specifically address the role of PKP4 in the intact epidermis. Second, it will be interesting to assess how PKP4 affects epithelial function and AJ-mediated tension in simple epithelial cells. This will be of special interest in the context of apoptotic or pyroptotic cell extrusion. It has been shown that contractility is essential for the expulsion of apoptotic cells which become hyper contractile to generate a tensile signal that is necessary to activate RhoA in its neighbors [84]. Finally, it will be important to analyze if PKP4 is involved in cytoskeletal and AJ alterations occurring in infectious and autoimmune diseases and in cancer.

### Supplementary Information

Below is the link to the electronic supplementary material.

Supplementary file1 (DOCX 2613 KB)

## Data Availability

RNA-seq data have been deposited at Gene Expression Omnibus (GEO) and are publicly available as of the date of publication. They were deposited into GSE255172 in GEO. Any additional data reported in this paper and any additional information required to reanalyze the data reported in this paper is available from the corresponding author upon request.

## Abbreviations

AJs: Adherens junctions
p120: P120-catenin
PKP4: Plakophilin 4
GEFs: Guanine nucleotide exchange factors
GAPs: GTPase-activating proteins
Rac1: Rac family small GTPase 1
Cdc42: Cell division cycle 42
Ect2: Epithelial cell transforming 2
FMRP: Fragile X mental retardation protein
ROCK: Ho associated kinase
MLC: Myosin regulatory light chains
LIMK: LIM kinase
F-actin: Filamentous actin
MMRRC: Mutant Mouse Resource and Research Center
PBS: Phosphate buffered saline
WT: Wildtype
KO: Knockout
FCS: Fetal calf serum
LCM: Low calcium medium
HCM: High calcium medium
EGTA: Ethylene glycol-bis-(2-aminoethylether)-N,N,N′,N′-tetraacetic acid
SDS: Sodium dodecyl sulfate
IP: Immunoprecipitation
TBST: Tris-buffered saline with Tween20
BSA: Bovine serum albumin
GAPDH: Glyceraldehyde-3-phosphate dehydrogenase
DABCO: 1,4- Diazabicyclo[2.2.2]octane
FDR: False discovery rate
GSEA: Gene set enrichment analysis
ANOVA: One-way analysis of variance
FAK: Focal adhesion kinase
MLCK: Myosin light chain kinase
MYPT: MLC phosphatase
ZA: Zonula adherens
GDI: Guanine nucleotide dissociation inhibitors
PF: Pemphigus Foliaceus
ARVCF: Armadillo repeat gene deleted in velo-cardio-facial syndrome
