# Supplementary material for: Plakophilin 4 controls the spatio-temporal activity of RhoA at adherens junctions to promote cortical actin ring formation and tissue tension
Source: Cell Mol Life Sci. 2024 Jul 6;81(1):291. doi: 10.1007/s00018-024-05329-6 (PMC11335210; doi:10.1007/s00018-024-05329-6)
Supplement: Supplementary file 1 — Supplementary file1 (DOCX 2613 KB) [file 18_2024_5329_MOESM1_ESM.docx]

SUPPLEMENTARY INFORMATION

**Plakophilin 4 controls the spatio-temporal activity of RhoA at adherens junctions to promote cortical actin ring formation and tissue tension**

Journal: Cellular and Molecuar Life Sciences

Lisa Müller^1,,*^, René Keil^1^, Markus Glaß^1^, and Mechthild Hatzfeld^1^

^1^ Institute of Molecular Medicine, Martin Luther University Halle-Wittenberg, Charles Tanford Protein Research Center, Kurt-Mothes-Str. 3A, 06120 Halle, Germany

* Correspondence: Lisa Müller, lisa.mueller@uk-halle.de


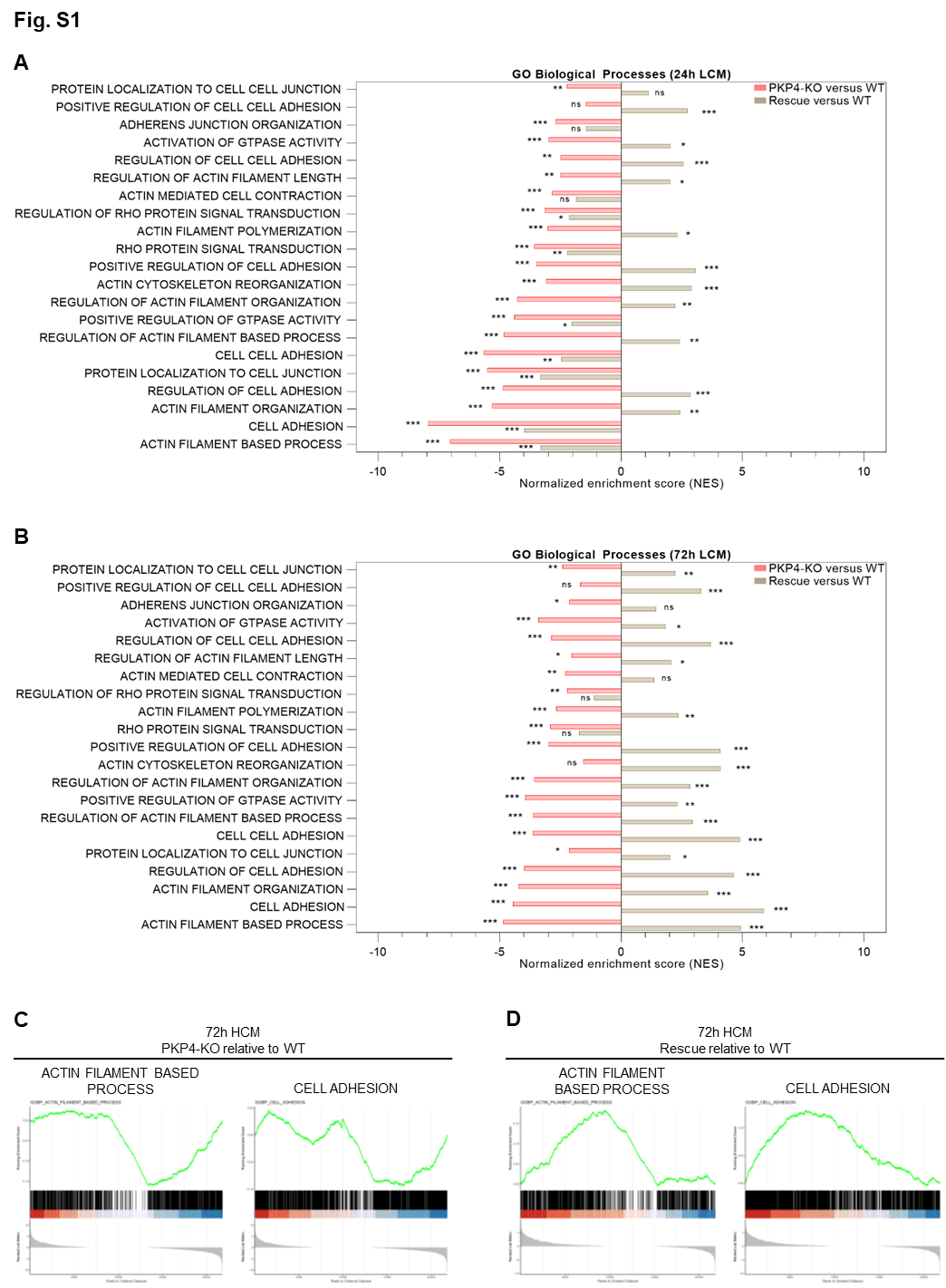


**Fig. S1: PKP4 regulates actin- and adhesion-associated pathways**

(A), (B) Normalized enrichment scores of selected gene sets among protein coding genes in PKP4-KO or Rescue (PKP4-KO+PKP4) versus WT cells after (A) 24h LCM or (B) 72h LCM. Positive values represent up regulation, negative values represent down regulation. Corresponding to Figure 1A and Figure 1B.

(C), (D) Enrichment plots for the two most significantly enriched GOBP gene sets for (C) PKP4-KO relative to WT cells or (D) Rescue (PKP4-KO+PKP4) relative to WT cells after 72h HCM, respectively. The plots show the profile of the running enrichment scores and positions of gene set members on the rank-ordered list. Genes on the far left (red) correspond to the most upregulated actin- or adhesion-associated genes, whereas genes on the far right (blue) correspond to the most downregulated actin- or adhesion-associated genes. Corresponding to Figure 1C and Figure 1D.

*P<0.05; **P<0.01; ***P<0.001; ns, not significant.


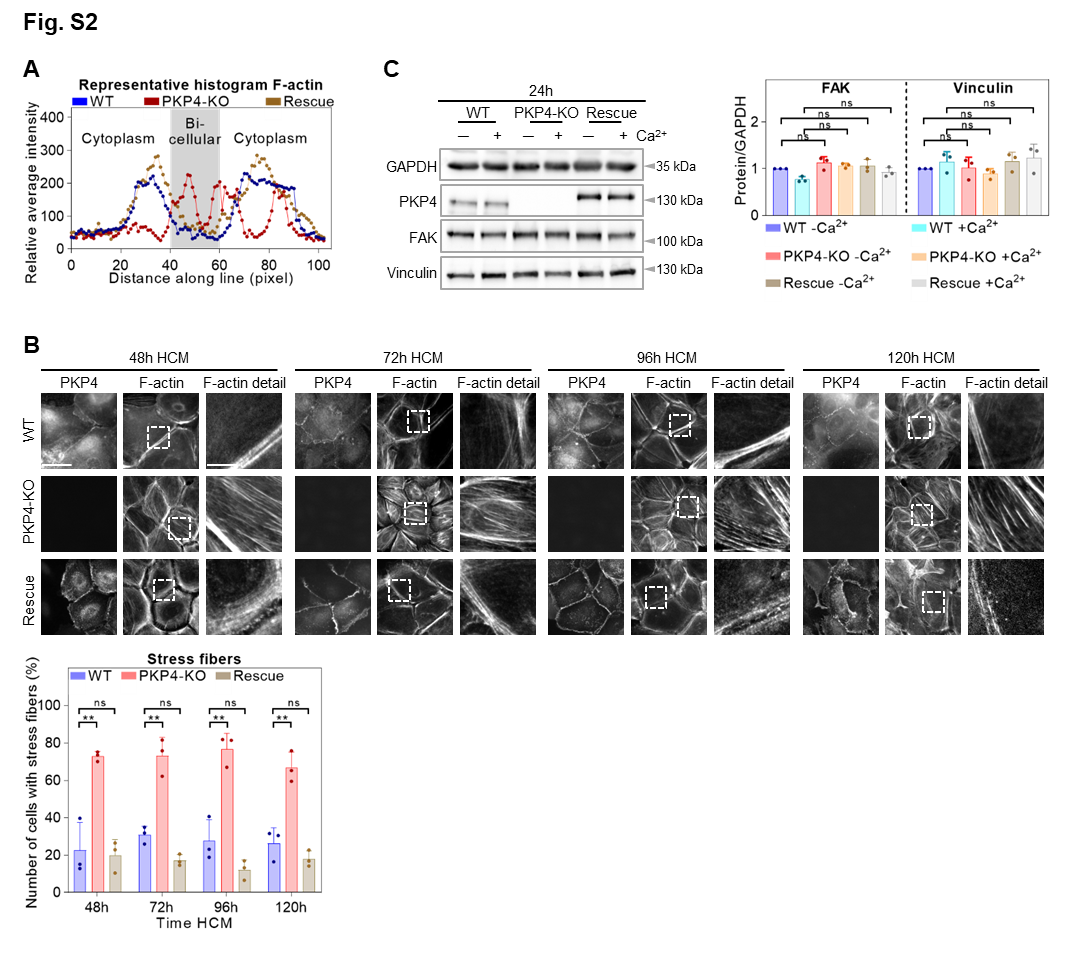


**Fig. S2: PKP4 affects actin organization**

(A) Representative histogram of relative average intensities of lateral F-actin in WT, PKP4-KO, and Rescue cells. Corresponding to Figure 2B.

(B) Immunofluorescence analysis of F-actin localization. Top: Representative immunofluorescence images showing PKP4 and F-actin localization in WT, PKP4-KO, and Rescue (PKP4-KO+PKP4) cells grown for 48h, 72, 96, or 120h in HCM. Scale bar = 50 µm, detail 10 µm. Bottom: Number of cells with stress fibers. Averages + SD from three independent experiments are plotted. n ≥ 100 cells per condition. Corresponding to Figure 2B.

(C) Levels of focal adhesion proteins in WT, PKP4-KO, and Rescue (PKP4-KO+PKP4) cells grown for 24h in medium with or without Ca^2+^. Left: Representative western blots of PKP4 and focal adhesion proteins. GAPDH was used as a loading control. Right: Quantification of protein amounts normalized to GAPDH and relative to WT cells grown in medium without Ca^2+^. Averages + SD from three independent experiments are plotted. Corresponding to Figure 2.

*P<0.05; **P<0.01; ***P<0.001; ns, not significant. Significance was determined by one-way ANOVA with Tukey’s multiple comparisons test (B, C).


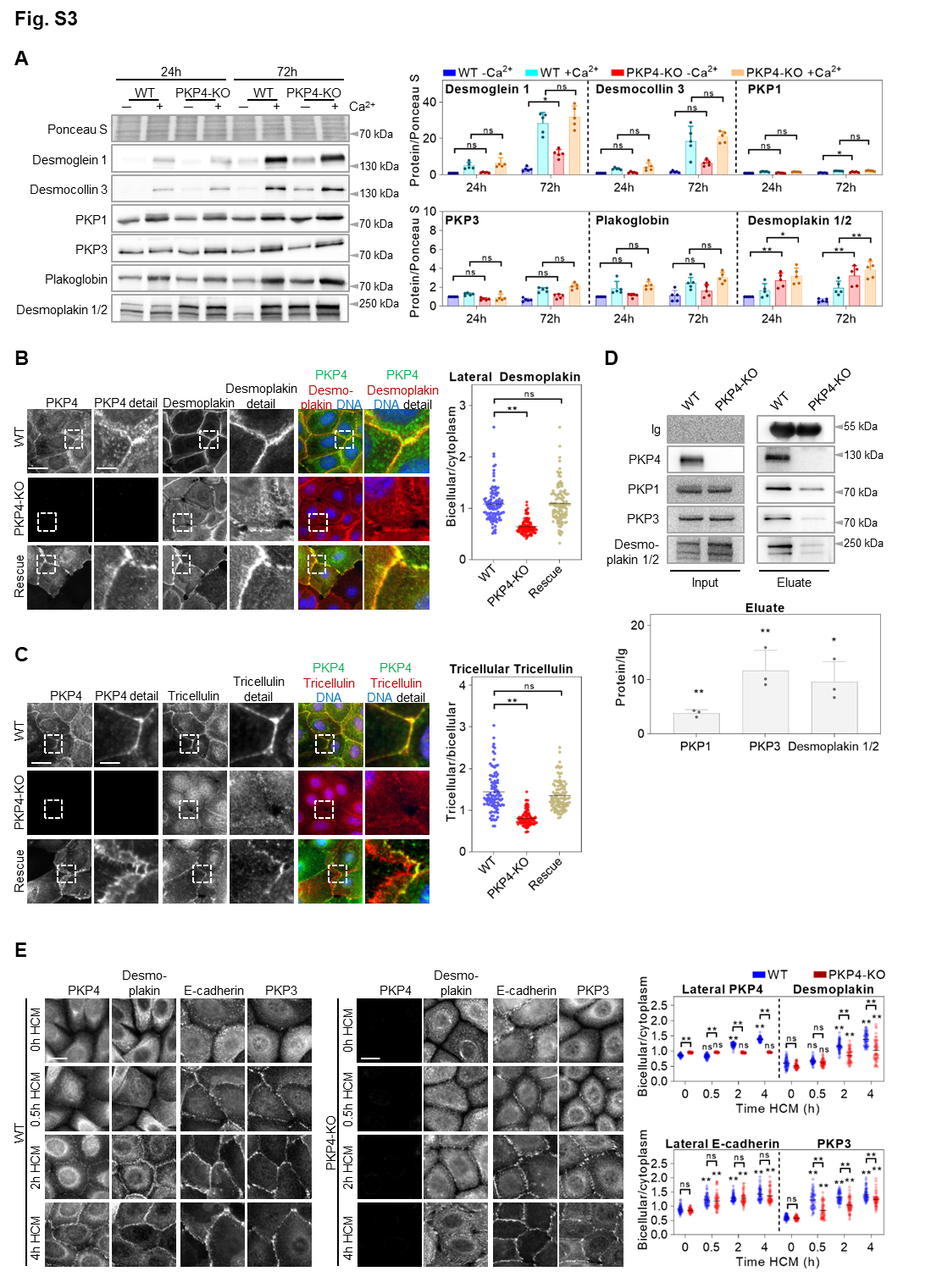


**Fig. S3: PKP4 modulates the morphology of junctions and stabilizes keratinocyte cell-cell adhesion**

(A) Levels of desmosomal proteins in WT and PKP4-KO cells grown for 24h or 72h in medium with or without Ca^2+^. Left: Representative western blots of desmosomal proteins. Ponceau S staining was used as a loading control. Right: Quantification of protein amounts normalized to Ponceau S staining and relative to WT cells grown for 24h in medium without Ca^2+^. Averages + SD from five independent experiments are plotted.

(B) Immunofluorescence analysis of the junctional localization of desmoplakin. Left: Representative immunofluorescence images showing PKP4 and DSP localization in WT, PKP4-KO, and Rescue (PKP4-KO+PKP4) cells. Scale bar = 50 µm, detail 10 µm. Right: Bicellular/cytoplasm or tricellular/bicellular ratio of desmoplakin fluorescence intensity. n ≥ 100 cells per condition from two independent experiments.

(C) Immunofluorescence analysis of the junctional localization of Tricellulin. Left: Representative immunofluorescence images showing PKP4 and Tricellulin localization in WT, PKP4-KO, and Rescue (PKP4-KO+PKP4) cells. Scale bar = 50 µm, detail 10 µm. Right: Tricellular/bicellular ratio of Tricellulin fluorescence intensity. n ≥ 100 cells per condition from two independent experiments.

(D) Endogenous PKP4 was affinity-purified from WT cells. PKP4-KO cells were treated in parallel as control. Top: Representative western blots of input and co-purifying proteins. Bottom: Enrichment of PKP1, PKP3, and desmoplakin normalized to Ig and relative to values of PKP4-KO cells (second lane in immunoblot, which was set to 1). Average + SD from three independent experiments was plotted.

(E) Immunofluorescence analysis of desmosome formation. Left: Representative immunofluorescence images showing PKP4, desmoplakin, E-cadherin, and PKP3 localization in WT and PKP4-KO cells incubated in HCM for the indicated time. Scale bar = 20 µm. Right: Bicellular/cytoplasm ratio of fluorescence intensity. n ≥ 50 cells per condition from two independent experiments.

*P<0.05; **P<0.01; ***P<0.001; ns, not significant. Significance was determined by one-way ANOVA with Tukey’s multiple comparisons test (A, B, C, E) or by student’s unpaired two tailed t-test (D). Corresponding to Figure 3.


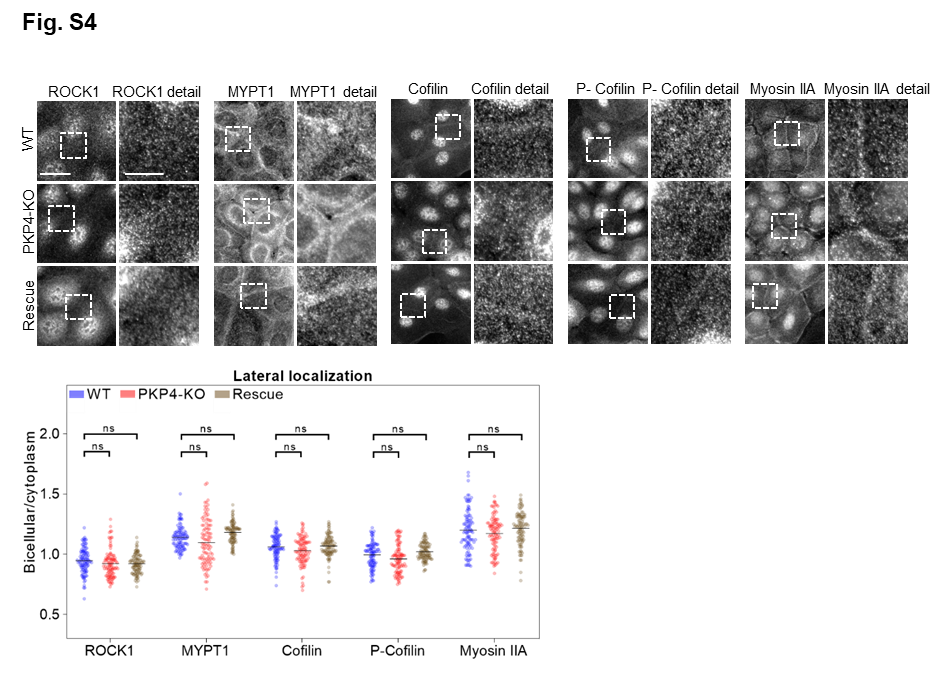


**Fig. S4: PKP4 promotes ROCK-signaling**

Immunofluorescence analysis of the junctional localization of proteins involved in ROCK-signaling. Top: Representative immunofluorescence images showing ROCK1, MYPT1, Cofilin, P-Cofilin-Ser3, and Myosin IIa localization in WT, PKP4-KO, and Rescue (PKP4-KO+PKP4) cells. Scale bar = 50 µm, detail 10 µm. Bottom: Bicellular/cytoplasm ratio of fluorescence intensities. n ≥ 100 cells per condition from two independent experiments. Corresponding to Figure 4C.

*P<0.05; **P<0.01; ***P<0.001; ns, not significant. Significance was determined by one-way ANOVA with Tukey’s multiple comparisons test.


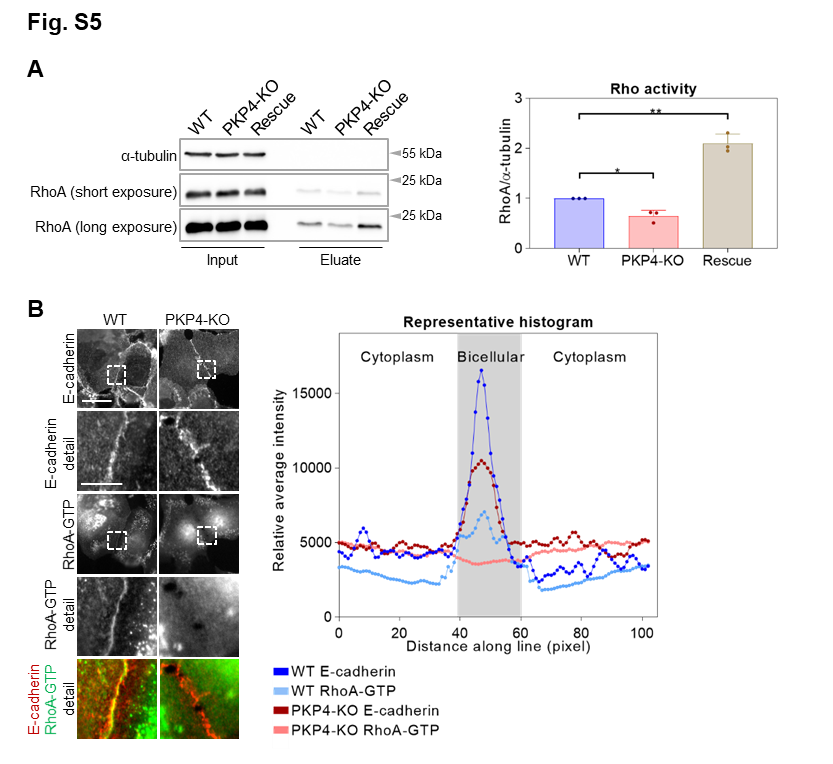


**Fig. S5: PKP4 promotes RhoA activation at the cell cortex and cortical actin ring formation**

(A) RhoA activity assay in WT, PKP4-KO, and Rescue (PKP4-KO+PKP4) cells. Left: Representative western blot of RhoA. α-tubulin was used as a loading control. Right: Enrichment of active RhoA normalized to input α-tubulin and relative to values of WT cells. Average + SD from three independent experiments was plotted. Corresponding to Figure 5.

(B) Immunofluorescence analysis of E-cadherin localization and the RhoA-GTP biosensor. Left: Representative immunofluorescence images showing E-cadherin and EGFP-RhoA biosensor localization in WT and PKP4-KO cells. Scale bar = 50 µm, detail 10 µm. Right: Histogram of the relative average intensities of lateral E-cadherin and RhoA-GTP. Averages ± SD from n ≥ 50 cells per condition from two independent experiments are plotted. Corresponding to Figure 5E.

*P<0.05; **P<0.01; ***P<0.001; ns, not significant. Significance was determined by one-way ANOVA with Tukey’s multiple comparisons test (A).


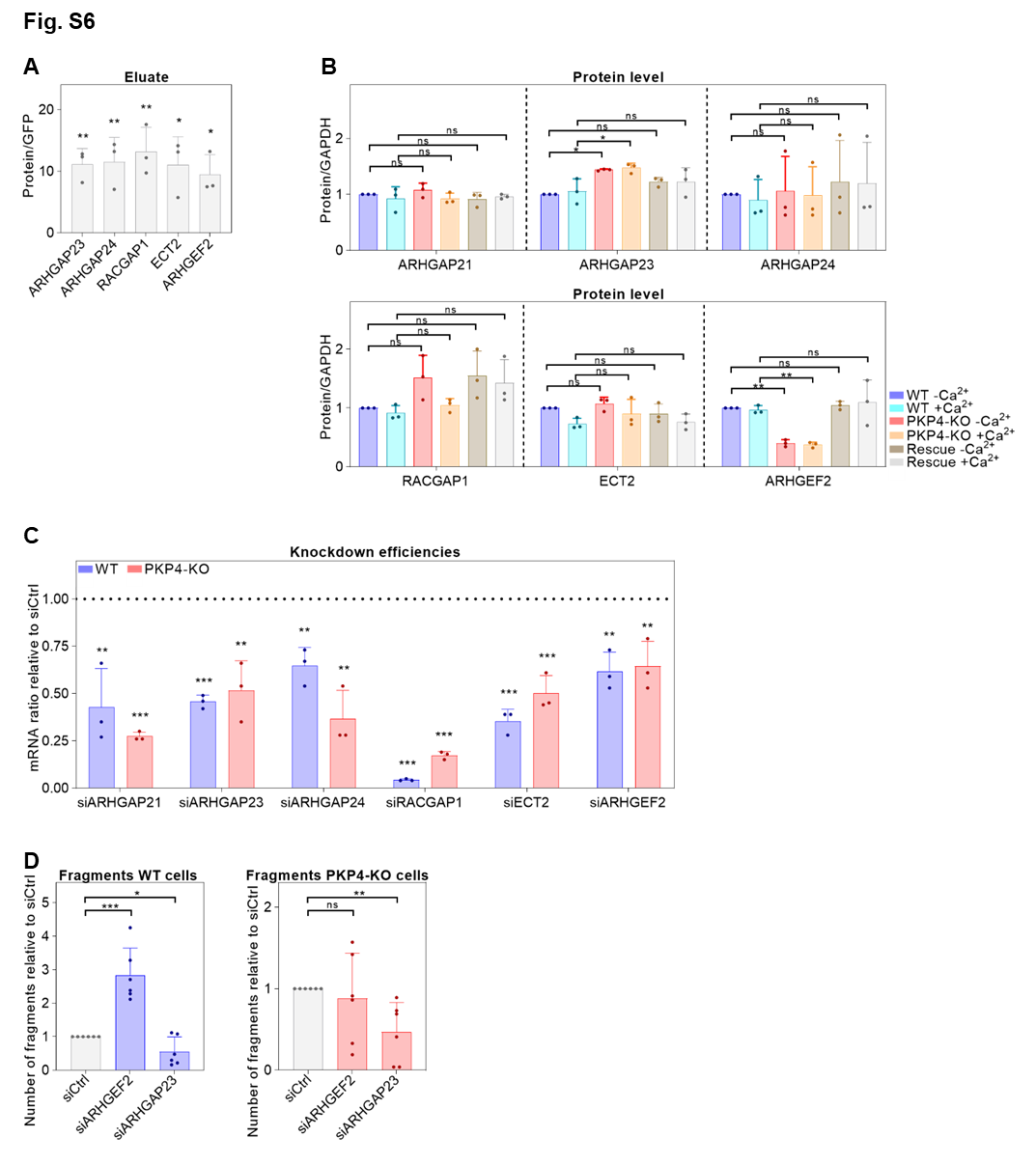


**Fig. S6: PKP4-dependent actin dynamics and tension are regulated by ARHGAP23 and ARHGEF2**

(A) GFP-PKP4 or GFP was affinity-purified from ectopically expressing WT or PKP4-KO cells, corresponding to Figure 6B. Enrichment of GEFs and GAPs normalized to precipitated GFP and relative to values of GFP cells (second lane in immunoblot, which was set to 1). Average + SD from three independent experiments was plotted. Significance was determined by student’s unpaired two-tailed t-test.

(B) Quantification of GEFs and GAPs protein amounts normalized to GAPDH and relative to WT cells grown in medium without Ca^2+^. Averages + SD from three independent experiments are plotted. Corresponding to Figure 6C.

(C) Validation of knockdown analysis in WT and PKP4-KO cells. Amounts of mRNA relative to non-targeting siRNA (siCtrl) treated cells. Averages + SD from three independent experiments are plotted. Corresponding to Figure 6D.

(D) Dispase-based dissociation assay of WT and PKP4-KO cells treated with ARHGEF2- or ARHGAP23-directed siRNAs and grown for 24h in HCM. Quantification of number of fragemnts relative to non-targeting siRNA (siCtrl) treated cells. Averages + SD from six independent experiments are plotted. Corresponding to Figure 6.

*P<0.05; **P<0.01; ***P<0.001; ns, not significant. Significance was determined by one-way ANOVA with Tukey’s multiple comparisons test (B) or by student’s unpaired two tailed t-test (A, C, D).

**Table S1: List of used siPools (defined pools of 30 selected siRNAs) obtained from siTools Biotech GmbH.**

| **Target** | **NCBI Gene ID** |
| --- | --- |
| non-targeting control | N/A |
| murine *ARHGAP21* | 71435 |
| murine *ARHGAP23* | 58996 |
| murine *ARHGAP24* | 231532 |
| murine *RACGAP1* | 26934 |
| murine *ECT2* | 13605 |
| murine *ARHGEF2* | 16800 |

**Table S2: List of primary antibodies, including source, identifier, and dilutions used in western blotting (WB) and immunofluorescence (IF).**

| Antibody | Source | Identifier | Dilution |
| --- | --- | --- | --- |
| Active Cdc42 mouse monoclonal antibody | Biomol | Cat# NB-26905 | 1:50 (IF) |
| Active Rac1 mouse monoclonal antibody | Biomol | Cat# NB-26903 | 1:200 (IF) |
| Active RhoA mouse monoclonal antibody | Biomol | Cat# NB-26904 | 1:500 (IF) |
| ARHGAP21 mouse monoclonal antibody | Santa Cruz Biotechnology | Cat# sc-390145 | 1:250 (WB) |
| ARHGAP23 rabbit polyclonal antibody | Affinity Biosiences | Cat# DF9857 | 1:50 (IF)  1:250 (WB) |
| ARHGAP24 rabbit polyclonal antibody | Affinity Biosiences | Cat# DF9858 | 1:250 (WB) |
| ARHGEF2 rabbit polyclonal antibody | Thermo Fisher Scientific | Cat# PA5-32213; RRID: 2549686 | 1:100 (IF)  1:250 (WB) |
| Cdc42 mouse monoclonal antibody | BD Biosciences | Cat# 610928; RRID: AB_398244 | 1:50 (IF) |
| Cofilin rabbit polyclonal antibody | Cell Signaling Technology | Cat# 3312 | 1:50 (IF)  1:500 (WB) |
| Desmocolin 3 mouse monoclonal antibody | Progen | Cat# 61093 | 1:1,000 (WB) |
| Desmoglein 1 mouse monoclonal antibody | Progen | Cat# 651111 | 1:1,000 (WB) |
| Desmoplakin 1/2 rabbit polyclonal antibody | Hatzfeld lab [38] | N/A | 1:500 (IF)  1:1,000 (WB) |
| E-cadherin rat monoclonal antibody | Thermo Fisher Scientific | Cat# 13-1900; RRID: AB_2533005 | 1:200 (IF)  1:500 (WB) |
| ECT2 rabbit monoclonal antibody | Cell Signaling Technology | Cat# 52021 | 1:250 (WB) |
| FAK goat polyclonal antibody | Santa Cruz Biotechnology | Cat# sc-557 | 1:50 (IF)  1:250 (WB) |
| GAPDH mouse monoclonal antibody | Sigma-Aldrich | Cat# 8795 | 1:1,000 (WB) |
| GFP rabbit polyclonal antibody | Rockland | Cat# 600-401-215; RRID: AB_828167 | 1:2,000 (WB) |
| MLC2 rabbit polyclonal antibody | Cell Signaling Technology | Cat# 3672 | 1:50 (IF)  1:500 (WB) |
| MLCK mouse monoclonal antibody | Santa Cruz Biotechnology | Cat #sc-58803 | 1:20 (IF)  1.100 (WB) |
| Myosin IIA rabbit polyclonal antibody | Cell Signaling Technology | Cat# 3403 | 1:50 (IF)  1:500 (WB) |
| MYPT1 rabbit polyclonal antibody | Santa Cruz Biotechnology | Cat# sc-25618 | 1:20 (IF)  1:100 (WB) |
| p120 mouse monoclonal antibody | BD Biosciences | Cat# 610134; RRID: AB_397537 | 1:500 (WB) |
| P-Cofilin-Ser3 rabbit monoclonal antibody | Cell Signaling Technology | Cat# 3313 | 1:50 (IF)  1:500 (WB) |
| P-MLC2-Ser19 rabbit polyclonal antibody | Cell Signaling Technology | Cat# 3671 | 1:50 (IF)  1:500 (WB) |
| PKP1 mouse monoclonal antibody | Santa Cruz Biotechnology | Cat# sc-33636 | 1:500 (WB) |
| PKP3 guinea pig polyclonal customized peptide specific antibody | Peptide Specialty Laboratories | N/A | 1:10,000 (IF)  1:20,000 (WB) |
| PKP4 mouse monoclonal antibody | Progen | Cat# 651166 | 1:10 (IF)  1:25 (WB) |
| Plakoglobin mouse monoclonal antbody | Progen | Cat# 61005 | 1:1,000 (WB) |
| Rac1 mouse monoclonal antbody | BD Biosciences | Cat# 610650; RRID: AB_397978 | 1:50 (IF) |
| RACGAP1 rabbit polyclonal antibody | Thermo Fisher Scientific | Cat# PA5-22265; RRID: AB_ 11155250 | 1:250 (WB) |
| RhoA rabbit rabbit monoclonal antibody | Cell Signaling Technology | Cat# 2117 | 1:50 (IF)  1:500 (WB) |
| ROCK1 mouse monoclonal antibody | BD Biosciences | Cat# 611136; RRID: AB_398448 | 1:50 (IF)  1:500 (WB) |
| ROCK2 mouse monoclonal antibody | BD Biosciences | Cat# 610623; RRID: AB_397956 | 1:50 (IF)  1:500 (WB) |
| Tricellulin rabbit polyclonal antibody | Thermo Fisher Scientific | Cat# 48-8400; RRID: AB_2533851 | 1:50 (IF) |
| Vinculin mouse monoclonal antibody | Sigma-Aldrich | Cat# MA5-11690; RRID: AB­_10976821 | 1:250 (IF)  1:500 (WB) |
| α-tubulin mouse monoclonal antibody | Sigma-Aldrich | Cat# T9026 | 1:1,000 (WB) |
| β-catenin mouse monoclonal antibody | BD Biosciences | Cat# 13-8400; RRID: AB_397555 | 1:1,000 (WB) |

**Table S3: List of secondary antibodies, including source, identifier, and dilutions used in western blotting (WB) and immunofluorescence (IF).**

| Antibody | Source | Identifier | Dilution |
| --- | --- | --- | --- |
| Peroxidase AffiniPure Donkey Anti-Goat IgG | Jackson ImmunoResearch | Cat# 705-035-003; RRID: AB_2340390 | 1:20,000 (WB) |
| Peroxidase AffiniPure Donkey Anti-Guinea Pig IgG | Jackson ImmunoResearch | Cat# 706-035-148; RRID: AB_2340447 | 1:15,000 (WB) |
| Peroxidase AffiniPure Donkey Anti-Mouse IgG | Jackson ImmunoResearch | Cat# 715-035-150; RRID: AB_2340770 | 1:20,000 (WB) |
| Peroxidase AffiniPure Donkey Anti-Rabbit IgG | Jackson ImmunoResearch | Cat# 711-035-152; RRID: AB_10015282 | 1:40,000 (WB) |
| Peroxidase AffiniPure Donkey Anti-Rat IgG | Jackson ImmunoResearch | Cat# 712-035-153; RRID: AB_2340639 | 1:20,000 (WB) |
| Alexa Fluor® 488 AffiniPure F(ab')₂ Fragment Donkey Anti-Mouse IgG | Jackson ImmunoResearch | Cat# 715-546-150; RRID: AB_2340849 | 1:400 (IF) |
| Alexa Fluor® 488 AffiniPure F(ab')₂ Fragment Donkey Anti-Rat IgG | Jackson ImmunoResearch | Cat# 715-546-153; RRID: AB_2340686 | 1:400 (IF) |
| Cy™3 AffiniPure F(ab')₂ Fragment Donkey Anti-Rabbit IgG | Jackson ImmunoResearch | Cat# 711-166-152, RRID: AB_2313568 | 1:200 (IF) |
| Cy™3 AffiniPure F(ab')₂ Fragment Donkey Anti-Rat IgG | Jackson ImmunoResearch | Cat# 712-166-153; RRID: AB_2340669 | 1:200 (IF) |
| Cy™3 AffiniPure F(ab')₂ Fragment Donkey Anti-Mouse IgG | Jackson ImmunoResearch | Cat# 715-166-151; RRID: AB_2340817 | 1:200 (IF) |
| Cy™3 AffiniPure F(ab')₂ Fragment Donkey Anti-Goat IgG | Jackson ImmunoResearch | Cat# 705-166-147; RRID: AB_2340413 | 1:200 (IF) |
| Cy™3 AffiniPure F(ab')₂ Fragment Donkey Anti-Guinea pig IgG | Jackson ImmunoResearch | Cat# 705-166-148; RRID: AB_2340461 | 1:200 (IF) |
| Alexa Fluor™ 568 Phalloidin | Thermo Fisher Scientific | Cat# A12380 | 1:400 (IF) |

**Table S4: List of primer pair sequences used in qRT-PCR.**

| Primer | Sequence 5’ -> 3’ |
| --- | --- |
| ARHGAP21-mouse_fw | GGCTGGCTTATGCACAGGTGA |
| ARHGAP21-mouse_rev | TGCCAGTGCTGTAACATCCTTTG |
| ARHGAP23-mouse_fw | CTTCTTCACGGACGGGTCTCT |
| ARHGAP23-mouse_rev | TAGACGCGCTTCCACTGGC |
| ARHGAP24-mouse_fw | AAGGCGGCTTTGTCAAGACT |
| ARHGAP24-mouse_rev | ATCTCGTTCACCTCCTGGAAC |
| ARHGEF2-mouse_fw | AGAGCCCCAGAGTGGTAAAC |
| ARHGEF2-mouse_rev | CCGTTGGTATAGCGGGCATC |
| ECT2-mouse_fw | ATCTCTGAGCGTGCCTTCATC |
| ECT2-mouse_rev | CACTCTTGCTTCAACCTGCGG |
| EIF3K_mouse_fw | TCGACAGGTACCAGTTCAACCC |
| EIF3K_mouse_rew | GCCGCTCTTCTTGATGTGCC |
| RACGAP1-mouse_fw | CGCGGGAAGTCAGGACCTTTA |
| RACGAP1-mouse_rev | CCGCTTTCCACACGGAACAC |

fw = forward, rev = reverse.
